# Baseline Body Mass Index and Anthropometric Responses to an Immersive Virtual Reality-Based Exercise Intervention: An Exploratory Secondary Analysis of a Randomized Controlled Trial

**DOI:** 10.3390/healthcare14183073

**Published:** 2026-09-18

**Authors:** Frano Giakoni-Ramírez, Andrés Godoy-Cumillaf, Josivaldo de Souza-Lima, Catalina Muñoz-Strale, Maribel Parra-Saldias, Daniel Duclos-Bastias, Claudio Farias-Valenzuela, Eugenio Merellano-Navarro, José Bruneau-Chávez

**Affiliations:** 1 Facultad de Educación y Ciencias Sociales, Instituto del Deporte y Bienestar, Universidad Andres Bello, Las Condes, Santiago 7550000, Chile; frano.giakoni@unab.cl (F.G.-R.); josivaldo.desouza@unab.cl (J.d.S.-L.); catalina.munoz@unab.cl (C.M.-S.); 2 Grupo de Investigación en Educación Física, Salud y Calidad de Vida (EFISAL), Facultad de Educación, Universidad Autónoma de Chile, Temuco 4780000, Chile; 3 Departamento de Educación Física, Deporte y Recreación, Universidad de Atacama, Copiapó 1530000, Chile; maribel.parra@uda.cl; 4 iGEO, Escuela de Educación Física, Facultad de Filosofía y Educación, Pontificia Universidad Católica de Valparaíso, Viña del Mar 2340025, Chile; daniel.duclos@pucv.cl; 5 METIS Research Lab, Facultad de Negocios y Tecnología, Universidad Alfonso X el Sabio (UAX), 28691 Madrid, Spain; 6 Escuela de Ciencias de la Actividad Física, Universidad de Las Américas, Santiago 8320000, Chile; claudio.farias.valenzuela@edu.udla.cl; 7 Department of Physical Activity Sciences, Faculty of Education Sciences, Universidad Católica del Maule, Talca 3530000, Chile; emerellano@ucm.cl; 8 Departamento de Educación Física, Deportes y Recreación, Universidad de la Frontera, Temuco 4811230, Chile; jose.bruneau@ufrontera.cl

**Keywords:** exergaming, digital health, body mass index, anthropometric, exercise intervention

## Abstract

**Highlights:**

**What are the main findings?**

- No statistically significant linear treatment-by-baseline body mass index (BMI) interaction was detected for post-intervention BMI, body weight, waist circumference, or hip circumference.
- The principal interaction estimates for BMI suggested increasingly negative IVR–control differences with increasing baseline BMI, but uncertainty widened toward the upper end of the BMI distribution; post hoc categorical findings were considered hypothesis-generating rather than confirmatory.

**What are the implications of the main findings?**

- The absence of a statistically significant interaction should not be interpreted as evidence of equivalent anthropometric responses across baseline BMI levels, because the modest sample size and limited representation at higher BMI values preclude excluding potentially meaningful treatment effect heterogeneity.
- Future trials specifically designed and adequately powered to evaluate treatment effect modification, with sufficient representation across the BMI distribution, are needed to determine whether baseline BMI or other participant characteristics influence anthropometric responses to IVR-based exercise interventions.

**Abstract:**

**Background/Objectives**: Whether baseline body mass index (BMI) modifies the anthropometric response to immersive virtual reality (IVR)-based exercise interventions remains unclear. This exploratory secondary analysis of a randomized controlled trial evaluated whether baseline BMI was associated with heterogeneity in the effect of an IVR-based exercise intervention on post-intervention anthropometric outcomes in university students. The treatment effect modification analysis was not prespecified in the parent trial protocol or registry. **Methods**: Sixty-four participants were randomized to IVR or control groups, of whom 60 (30 per group) provided post-intervention anthropometric data and were included in the principal complete-case analysis. Post-intervention BMI was designated as the principal outcome of the present secondary analysis, whereas body weight, waist circumference, and hip circumference were secondary outcomes. Treatment effect modification was evaluated using baseline-adjusted ordinary least-squares regression models including treatment allocation, centered baseline BMI, and the treatment × baseline BMI interaction. Sensitivity analyses included adjustment for sex, HC3 heteroscedasticity-consistent standard errors, influence diagnostics, exploratory assessment of nonlinearity, and multiple imputation including all 64 randomized participants. Categorical BMI analyses were conducted post hoc and considered exploratory. **Results**: No statistically significant linear treatment-by-baseline BMI interaction was detected for post-intervention BMI (B = −0.039, 95% CI: −0.098 to 0.020; *p* = 0.189) or for body weight, waist circumference, or hip circumference (all *p* > 0.05). Model-estimated IVR–control differences in post-intervention BMI became progressively more negative with increasing baseline BMI, but uncertainty widened toward the upper end of the observed BMI distribution. Multiple imputation including all 64 randomized participants yielded a nearly identical interaction estimate (B = −0.039, 95% CI: −0.097 to 0.020; *p* = 0.192). Post hoc categorical analyses yielded different patterns from the principal continuous analysis and were considered hypothesis-generating. **Conclusions**: No statistically significant linear treatment-by-baseline BMI interaction was detected for post-intervention BMI or the secondary anthropometric outcomes. However, the modest sample size, limited representation at the upper end of the BMI distribution and widening uncertainty in the conditional estimates preclude excluding potentially meaningful treatment effect heterogeneity. These exploratory findings should not be interpreted as evidence of equivalent anthropometric responses across baseline BMI levels and require confirmation in trials specifically designed and adequately powered to evaluate effect modification.

## 1. Introduction

Overweight and obesity remain among the most important public health challenges worldwide and are associated with an increased risk of cardiovascular disease, type 2 diabetes, several cancers, musculoskeletal disorders, and premature mortality [1,2]. Recent global estimates indicate that the prevalence of overweight and obesity has continued to increase over recent decades, reinforcing the need for effective and scalable intervention strategies [1]. Physical activity is a cornerstone of weight management because it contributes to energy balance and provides important cardiometabolic and functional health benefits [2,3].

Despite these well-established benefits, maintaining regular participation in physical activity remains challenging for many individuals with excess body weight. Adults with overweight or obesity frequently encounter barriers to exercise participation, including physical discomfort, reduced motivation, fear of negative judgment, and weight-related stigma, all of which may compromise long-term adherence to structured exercise programs [4,5,6]. Because adherence is a key determinant of the effectiveness of lifestyle interventions, identifying exercise modalities that promote sustained participation is an important consideration in intervention design [4,5].

Immersive virtual reality (IVR) has recently emerged as an innovative approach for delivering exercise through interactive and engaging environments. IVR-based exercise may provide an engaging alternative to conventional exercise modalities, with studies reporting favorable experiences related to enjoyment, motivation, and engagement, while also allowing participants to achieve exercise intensities capable of eliciting physiological adaptations [7,8,9,10]. Recent systematic reviews and meta-analyses have reported that IVR-based exercise is feasible across different populations and may contribute to improvements in physical activity participation and selected health-related outcomes, although evidence regarding anthropometric adaptations remains limited and heterogeneous [7,10,11].

Although IVR represents a promising strategy for promoting exercise participation, an important question remains insufficiently explored: whether anthropometric responses to IVR-based exercise vary according to baseline BMI. Individual responses to exercise interventions can vary substantially, and identifying participant characteristics associated with such variation may help characterize treatment effect heterogeneity and inform the design and interpretation of future interventions [12,13]. Baseline BMI is a readily available anthropometric characteristic that may be relevant in this context, particularly because individuals across the BMI distribution may differ in physiological characteristics and in barriers to exercise participation [4,5,6]. Importantly, the present analysis was not restricted to individuals with overweight or obesity; rather, baseline BMI was evaluated across its observed continuous distribution in a general university-student sample. However, evidence regarding baseline BMI as a potential treatment effect modifier of anthropometric responses to IVR-based exercise remains limited. Consequently, evaluating BMI within an interaction-based analytical framework may provide additional insight into whether the effect of IVR-based exercise varies according to participants’ baseline anthropometric status.

Accordingly, the aim of the present exploratory secondary analysis was to evaluate whether baseline BMI was associated with heterogeneity in the effect of an IVR-based exercise intervention on post-intervention anthropometric outcomes by formally testing treatment-by-baseline BMI interactions. This treatment effect modification analysis was not prespecified in the parent trial protocol or registry and should therefore be considered exploratory. Accordingly, no directional treatment effect modification hypothesis was prespecified regarding whether increasing baseline BMI would be associated with a greater or smaller anthropometric response to the intervention. Post-intervention BMI was designated as the principal outcome of the present secondary analysis, whereas body weight, waist circumference, and hip circumference were considered secondary outcomes. Baseline BMI was evaluated primarily as a continuous potential treatment effect modifier, while analyses using the conventional BMI threshold of 25 kg/m^2^ were conducted post hoc as exploratory complementary analyses.

## 2. Materials and Methods

### 2.1. Study Design

This study is an exploratory secondary analysis of data from a previously registered randomized controlled trial that evaluated the effects of an immersive virtual reality (IVR)-based exercise intervention in university students. The parent trial used a two-arm parallel-group design (IVR and control) with a 12-week intervention period.

A total of 64 participants were randomized in the parent trial (32 to IVR and 32 to control), all of whom had baseline anthropometric data available. Four participants (two in each group) voluntarily discontinued study participation and did not complete the post-intervention assessment. Accordingly, the principal complete-case analyses included the 60 participants with both baseline and post-intervention anthropometric data (30 IVR and 30 control). A multiple-imputation sensitivity analysis including all 64 randomized participants was additionally conducted to assess the potential influence of missing post-intervention BMI data.

Baseline BMI was evaluated primarily as a continuous treatment effect modifier. Although BMI was among the anthropometric outcomes assessed in the parent randomized controlled trial, treatment effect modification by baseline BMI was not prespecified in the original trial protocol or registry. Therefore, all moderation analyses reported in the present study should be considered exploratory rather than confirmatory.

Participant recruitment for the parent trial was conducted between May and July 2025, with baseline assessments performed during the final two weeks of July 2025. The 12-week intervention was conducted from August to October 2025, with post-intervention assessments performed following completion of the intervention period. The IVR-based exercise intervention consisted of three supervised exercise sessions per week using a standardized protocol.

The primary findings of the parent randomized controlled trial have been reported previously [14]. That report presented the average intervention effects on anthropometric outcomes, including body weight, BMI, waist circumference, and hip circumference, as well as physical fitness and blood pressure outcomes. Accordingly, some baseline and post-intervention descriptive anthropometric data derive from the same measurements previously reported in the parent trial. The present study uses data from the same randomized participants and anthropometric assessments to address a distinct secondary research question concerning treatment effect modification by baseline BMI; the treatment-by-baseline BMI interaction analyses, conditional treatment effect estimates, and associated sensitivity analyses reported here were not included in the primary report of the parent trial. The reporting of this exploratory secondary analysis was guided by the CONSORT 2025 statement, with particular attention to recommendations for reporting additional analyses, including treatment effect modification and sensitivity analyses [15].

### 2.2. Ethical Considerations

All study procedures were conducted in accordance with the ethical principles of the Declaration of Helsinki. The study protocol was reviewed and approved by the Scientific Ethics Committee of the Universidad Autónoma de Chile (Approval No. CEC 26-24). Written informed consent was obtained from all participants prior to enrollment. The informed consent form stated that the data collected would be used exclusively for scientific purposes and that the study findings would be disseminated through scientific publication. The Scientific Ethics Committee of the Universidad Autónoma de Chile was subsequently consulted regarding the use of the existing trial dataset for the present secondary analysis and confirmed that the analysis could be conducted. No new data were collected and no additional contact with participants occurred for the present secondary analysis. The present treatment effect modification analysis was not included in the original trial protocol or registry and is therefore reported as an exploratory, non-prespecified secondary analysis of the parent randomized controlled trial. The intervention was conducted as part of a registered clinical trial (ClinicalTrials.gov Identifier: NCT06580769), and the complete study protocol describing the intervention procedures has been published previously [16].

### 2.3. Participants

Participants were recruited from undergraduate programs at the Universidad Autónoma de Chile (Temuco campus, Chile) through announcements distributed via institutional e-mail, social media platforms, and informational posters displayed across the university campus. Eligible participants were undergraduate students aged 18–25 years who provided written informed consent and submitted a medical certificate authorizing participation in physical activity.

The inclusion criteria were: (1) enrollment as an undergraduate student at the Universidad Autónoma de Chile; (2) age between 18 and 25 years; (3) submission of a medical certificate authorizing participation in physical activity; and (4) provision of written informed consent. Exclusion criteria comprised: (1) any physical or mental condition that could preclude safe participation in the intervention; (2) visual impairments or medical conditions incompatible with the use of immersive virtual reality, including epilepsy, photosensitive disorders, vestibular dysfunction, recurrent dizziness, or a history of cybersickness; and (3) belonging to special population groups, such as professional athletes or pregnant women. Eligibility was verified before enrollment based on the medical certificate provided by each participant and the predefined inclusion and exclusion criteria.

The parent randomized controlled trial enrolled 64 participants, who were allocated in a 1:1 ratio to either the immersive virtual reality (IVR) intervention group or the control group (32 participants per group) through simple randomization using an electronic random draw generated with EPIDAT version 4.2 software. Further details regarding the implementation of allocation concealment were not documented in the available parent-trial protocol or primary report.

**Figure 1:**
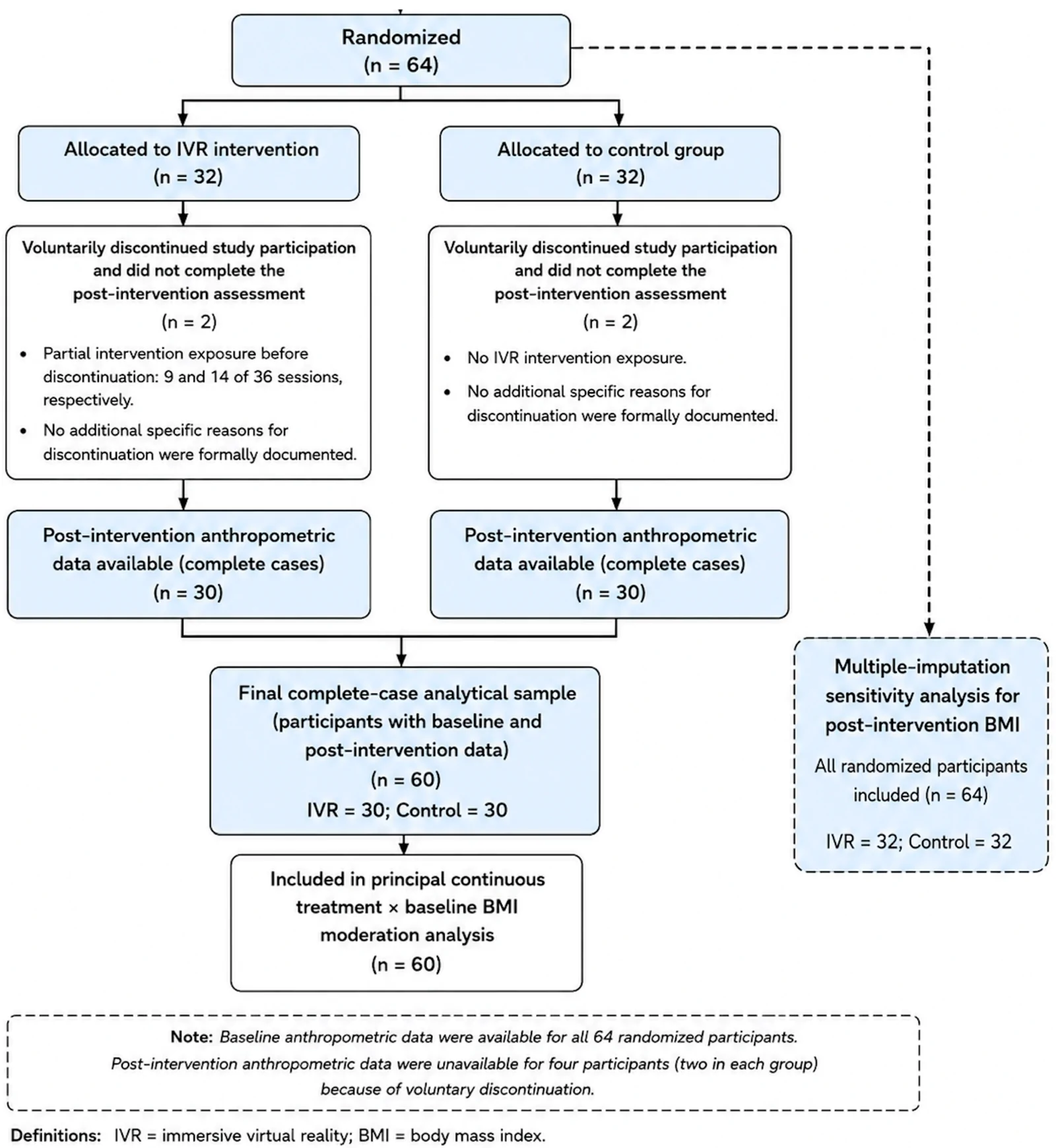
Participant flow from randomization through the principal complete-case analysis and multiple-imputation sensitivity analysis. The principal continuous treatment-by-baseline BMI moderation analysis included 60 participants with complete baseline and post-intervention anthropometric data. The multiple-imputation sensitivity analysis for post-intervention BMI included all 64 randomized participants. BMI, body mass index; IVR, immersive virtual reality.

Four participants (two from each group) voluntarily discontinued study participation and did not complete the post-intervention assessment. Baseline data were available for all four participants. These four participants were not included in the analytical sample of 60 participants reported in the published primary report of the parent trial [14]. The two participants allocated to the IVR group had partial exposure to the intervention before discontinuation, whereas the two participants allocated to the control group did not receive the IVR-based exercise intervention. No post-intervention anthropometric data were available for these four participants. Accordingly, the principal complete-case analysis included 60 participants (30 IVR and 30 control) with both baseline and post-intervention anthropometric assessments. As a sensitivity analysis addressing missing post-intervention BMI data, all 64 randomized participants were subsequently included using multiple imputation, as described in the Statistical Analysis section. The flow of participants from randomization in the parent trial to the principal complete-case analysis and the missing-data sensitivity analysis is shown in Figure 1. Exact counts of individuals assessed for eligibility and excluded before randomization, together with reasons for pre-randomization exclusion, could not be reconstructed from the parent-trial records available for the present secondary analysis; therefore, Figure 1 begins at randomization. All evaluations and intervention sessions were conducted at the Virtual Reality Laboratory of the Physical Education Pedagogy Program, Universidad Autónoma de Chile, Temuco campus.

### 2.4. Intervention and Control Condition

Participants allocated to the IVR group completed a supervised immersive virtual reality-based exercise program consisting of three training sessions per week over a 12-week period. All sessions were conducted individually at the Virtual Reality Laboratory of the Universidad Autónoma de Chile (Temuco campus, Chile) under the supervision of trained physical education professionals. Although the same professional did not necessarily supervise every session, all supervisors received prior training in the standardized intervention protocol and followed the same session structure, intensity targets, safety procedures, and criteria for adjusting exercise intensity.

The intervention was delivered using a head-mounted virtual reality headset (Meta Quest 2; Meta Platforms Inc., Menlo Park, CA, USA) and the FITXR application (version 3.7.88; FITXR Limited, London, UK). Only the Boxing mode was used throughout the intervention. Before each session, the headset was individually adjusted to ensure appropriate fit, comfort, and visual alignment. All participants followed the same standardized exercise protocol throughout the 12-week intervention.

Maximal heart rate was determined individually before the intervention through a maximal incremental cardiorespiratory exercise test supervised by a cardiologist. The test was performed on a Monark LC7TT cycle ergometer (Monark Exercise AB, Vansbro, Sweden), while heart rate was continuously recorded using a Polar H10 heart rate monitor (Polar Electro Oy, Kempele, Finland). After a 5 min unloaded warm-up, workload was increased by 25 W every 2 min while participants maintained a cadence of 60 rpm. The test was terminated when the participant was no longer able to maintain the prescribed cadence. The maximal heart rate recorded during the test was used to individualize exercise intensity during the IVR sessions.

Each training session lasted approximately 44 min and comprised a 5 min warm-up, a 30 min main exercise phase, two 2 min recovery periods between exercise blocks, and a 5 min cool-down. The warm-up was performed at moderate intensity (60–70% of maximal heart rate). The main exercise phase consisted of three consecutive 10 min Boxing blocks of increasing difficulty: beginner (Box Drills 18), intermediate (Rhythmic Burn), and advanced (Party Up), performed at vigorous intensity (approximately 80–95% of maximal heart rate). A 2 min recovery period was provided between consecutive exercise blocks to allow participants to rest, hydrate, and, when necessary, dry perspiration. The same three-block structure was used in every training session throughout the 12-week intervention. The session concluded with mobility and stretching exercises performed at low intensity (50–60% of maximal heart rate).

Heart rate was monitored throughout the IVR training sessions using Polar H10 monitors (Polar Electro Oy, Kempele, Finland). Supervising professionals used the real-time heart-rate response to verify whether participants were exercising within their individually prescribed intensity ranges and instructed participants to increase or decrease exercise intensity when necessary. Session-level heart-rate data were not retained for the present secondary analysis; therefore, the proportion of exercise time actually spent within the prescribed intensity ranges could not be quantified retrospectively.

To ensure participant safety, all sessions were performed in a clear exercise area of at least 3 m^2^ under continuous supervision. Participants were instructed to discontinue the session immediately if they experienced dizziness, nausea, visual discomfort, or any symptoms compatible with cybersickness. No adverse events or symptoms compatible with cybersickness requiring interruption or discontinuation of the intervention were observed among the 30 IVR participants who completed the post-intervention assessment. The two IVR participants who discontinued study participation had attended 9 and 14 sessions, respectively, before discontinuation; their discontinuation was not documented as being related to injury, and no additional specific reasons were formally documented. Complete adverse-event information was not available for these two participants; therefore, the absence of adverse events cannot be confirmed for all participants allocated to the IVR group. Participants were also instructed to maintain their usual dietary habits throughout the intervention and to avoid engaging in additional structured exercise programs during the study period. No formal dietary monitoring or dietary control was performed during the intervention period. Physical activity or exercise performed outside the study intervention was not formally monitored in either group.

Participants allocated to the control group were instructed to continue their usual physical activity throughout the 12-week study period. They received general information once weekly regarding the cardiovascular health benefits of physical exercise through informative brochures distributed via social networks. The educational content described examples of activities such as walking, playing, dancing, and fitness and their potential contribution to health. The control group did not receive the structured IVR-based exercise intervention.

Adherence to the intervention was defined a priori in the parent trial protocol as attendance at ≥70% of the scheduled training sessions [16]. All 30 IVR participants with available post-intervention data were included in the principal complete-case analyses irrespective of whether they met this adherence criterion. Among these participants, mean attendance was 27.5 ± 1.9 of 36 scheduled sessions (76.3%), with a median of 27.5 sessions and a range of 25–31 sessions. Twenty-two participants (73.3%) attended ≥70% of the scheduled sessions. The remaining eight participants (26.7%) each attended 25 of 36 sessions (69.4%), corresponding to one session below the protocol-defined adherence threshold. Thus, among the 30 IVR participants included in the principal complete-case analysis, no participant attended fewer than 25 of the 36 scheduled sessions. The two IVR participants who discontinued before post-intervention assessment had attended 9 and 14 sessions, respectively, as described above.

### 2.5. Anthropometric Assessment

Anthropometric assessments were conducted between 08:00 and 09:00 h under standardized conditions by trained evaluators. Participants were assessed after an overnight fast of approximately 8–10 h and were instructed to maintain their usual hydration status and to empty their bladder before the measurements. Assessments were performed with participants barefoot and wearing minimal lightweight sports clothing. Evaluators received specific training in the anthropometric procedures and followed a standardized measurement protocol. The same evaluator assessed each participant at baseline and post-intervention, and evaluators remained blinded to randomized treatment allocation during the post-intervention assessments. The success of assessor blinding was not formally evaluated.

Body weight was measured to the nearest 0.1 kg using a digital scale (Omron, Kyoto, Japan). Stature was measured to the nearest 0.1 cm using a portable stadiometer (Seca, Hamburg, Germany), with participants standing barefoot in an upright position with the head positioned in the Frankfort horizontal plane. Both measurements were obtained in duplicate, and the average value was used for subsequent analyses. Body weight and stature were measured at both baseline and post-intervention assessments. Body mass index (BMI) was calculated as body weight (kg) divided by the square of stature (m^2^) using the corresponding weight and stature measurements obtained at each assessment time point. No additional correction was applied for between-assessment variation in stature; potential measurement variability was addressed through duplicate measurements, standardized procedures, and use of the same evaluator at both assessments.

Waist circumference was measured at the midpoint between the lower margin of the last rib and the upper border of the iliac crest, whereas hip circumference was measured at the level of the maximum gluteal protuberance using a non-elastic anthropometric tape (Holway, San José, CA, USA). Circumference measurements were obtained in duplicate, and the mean value of the two measurements was used for statistical analyses. The same equipment and measurement procedures were used at baseline and post-intervention.

Formal equipment calibration records were not available for the present secondary analysis, and study-specific technical error of measurement (TEM) or intra- and inter-rater reliability coefficients were not calculated. To reduce measurement variability, all assessments followed standardized procedures, measurements were obtained in duplicate, evaluators received specific training, and the same evaluator assessed each participant at baseline and post-intervention.

### 2.6. Statistical Analysis

All statistical analyses reported in the revised manuscript were independently reproduced and verified using Python version 3.13.14. Data management was performed using pandas version 2.2.3 and NumPy version 2.3.5, regression analyses using statsmodels version 0.14.6, and additional statistical procedures using SciPy version 1.17.1. A reproducible Python script implementing the final analytical workflow is provided as Supplementary Code S1. NumPy and SciPy were additionally used to implement the Bayesian linear-regression multiple-imputation sensitivity analysis described below.

For the present secondary analysis, post-intervention BMI was selected as the principal outcome, whereas post-intervention body weight, waist circumference, and hip circumference were considered secondary outcomes. This outcome hierarchy was established during development of the analytical plan for the present secondary analysis, before inspection of the treatment-by-baseline BMI interaction results. Post-intervention BMI was selected as the principal outcome on conceptual grounds because it corresponded directly to the baseline BMI variable evaluated as the potential treatment effect modifier and represented the most direct outcome for addressing the central analytical question. This analytical hierarchy was not prespecified in the parent trial protocol or registry and does not confer confirmatory status on the moderation analyses. Accordingly, all treatment effect modification analyses reported in the present study were considered exploratory. Baseline BMI was centered at the overall sample mean to improve model interpretability. Treatment allocation was coded as 0 (control) and 1 (IVR).

The principal moderation analysis consisted of an analysis of covariance (ANCOVA) framework implemented using ordinary least-squares regression [17]. For post-intervention BMI, post-intervention BMI was entered as the dependent variable, while treatment allocation, centered baseline BMI, and the treatment × baseline BMI interaction were included as independent variables. The interaction term represented the principal test of linear treatment effect modification by baseline BMI. The treatment coefficient represented the adjusted IVR–control difference at the mean baseline BMI, whereas the centered BMI coefficient represented the association between baseline BMI and post-intervention BMI within the control group. To facilitate interpretation of the interaction estimate and its precision across the observed BMI distribution, model-derived IVR–control differences and corresponding 95% confidence intervals (CIs) were additionally estimated at baseline BMI values of 22, 27, 32, and 37 kg/m^2^. These values were selected to illustrate treatment effect estimates across the observed BMI range and were interpreted descriptively rather than as separate tests of effect modification.

For the secondary anthropometric outcomes (body weight, waist circumference, and hip circumference), separate ANCOVA models were fitted using the corresponding post-intervention value as the dependent variable. Each model included treatment allocation, centered baseline BMI, the treatment × baseline BMI interaction, and the corresponding baseline value of the outcome as a covariate. The three secondary interaction tests were treated as a single secondary family of exploratory hypotheses, and their *p*-values were adjusted using the Holm procedure [18]. The principal BMI interaction was reported with its unadjusted *p*-value and 95% CI in accordance with the outcome hierarchy established before inspection of the interaction results.

Continuous variables are presented as mean ± standard deviation (SD), whereas categorical variables are reported as frequencies and percentages. Baseline characteristics were summarized descriptively using standardized mean differences (SMDs), without between-group hypothesis testing. Statistical significance was established at a two-sided α level of 0.05. Given the non-prespecified and exploratory nature of the present moderation analysis, statistical significance was interpreted together with effect estimates and 95% CIs, and absence of statistical significance was not interpreted as evidence of equivalence or absence of treatment effect heterogeneity.

Because sex is a biologically plausible prognostic variable for anthropometric outcomes and its distribution also differed between randomized groups, a sensitivity analysis was performed by additionally adjusting the principal BMI moderation model for sex. Corresponding sex-adjusted sensitivity models were also fitted for body weight, waist circumference, and hip circumference. Sex was selected based on its prognostic relevance rather than solely on the magnitude of the observed baseline imbalance. These analyses were used to evaluate whether adjustment for sex materially altered the treatment × baseline BMI interaction estimates. Age and other baseline characteristics were not additionally included solely because of observed between-group imbalance, in order to avoid data-driven covariate selection and unnecessary expansion of the models given the modest sample size and exploratory nature of the analysis.

Model assumptions were evaluated by examining residual normality using the Shapiro–Wilk test, homoscedasticity using the Breusch–Pagan test, multicollinearity using variance inflation factors (VIF), and influential observations using Cook’s distance, leverage values, and externally studentized residuals. Sensitivity analyses were conducted using HC3 heteroscedasticity-consistent standard errors and by refitting the models after excluding observations exceeding the predefined Cook’s distance threshold of 4/N.

Potential non-linearity of the moderating effect of baseline BMI was explored by extending the linear interaction models with a quadratic BMI term and its interaction with treatment allocation. Model fit was compared using nested F-tests, Akaike information criterion (AIC), and adjusted coefficients of determination (adjusted R^2^). Linear models were retained as the principal analytical approach because of the modest sample size and to preserve model parsimony. The quadratic analyses were considered hypothesis-generating assessments of potential departures from linearity and were not used to make confirmatory inferences.

As an additional post hoc exploratory analysis, baseline BMI was categorized as <25 versus ≥25 kg/m^2^ using the conventional BMI threshold. Because this categorization was derived from the same continuous baseline BMI variable and was not prespecified in the parent trial protocol or registry, these analyses were considered hypothesis-generating and were interpreted separately from the principal continuous interaction analysis.

The principal analyses used a complete-case approach and included the 60 participants with observed baseline and post-intervention anthropometric data. Because baseline data were available for all 64 randomized participants but post-intervention anthropometric data were missing for four participants (two IVR and two control), a multiple-imputation sensitivity analysis was conducted for the principal BMI moderation model under a Missing At Random (MAR) assumption. Fifty imputed datasets were generated using Bayesian linear-regression imputation. The imputation model included treatment allocation, sex, baseline BMI, body weight, stature, waist circumference, hip circumference, and the fully observed treatment × baseline BMI product term to preserve compatibility with the substantive interaction model. For each imputation, regression coefficients and residual variance were drawn from their posterior distributions, and missing post-intervention BMI values were generated from the corresponding posterior predictive distribution. The substantive BMI moderation model was fitted separately in each completed dataset, and estimates were pooled using Rubin’s rules with Barnard–Rubin small-sample degrees of freedom. This analysis included all 64 randomized participants and was used solely to assess the sensitivity of the complete-case interaction estimate to missing post-intervention BMI data.

## 3. Results

### 3.1. Participant Characteristics

Table 1 presents the baseline characteristics of the 60 participants included in the principal complete-case analysis according to randomized treatment allocation (30 IVR and 30 control). The overall mean baseline BMI was 26.30 kg/m^2^, with observed values ranging from 19.9 to 39.1 kg/m^2^. Standardized differences in baseline anthropometric characteristics were small (absolute SMDs ≤ 0.15). Larger standardized differences were observed for sex (SMD = −0.42) and age (SMD = −0.31), with a lower proportion of women and a slightly lower mean age in the IVR group. Twenty-eight participants (46.7%) had BMI < 25 kg/m^2^, 24 (40.0%) had BMI between 25 and 29.9 kg/m^2^, and only eight (13.3%) had BMI ≥ 30 kg/m^2^; of the latter, two were allocated to IVR and six to control. Accordingly, interpretation of treatment effect modification is primarily supported within the more densely represented portion of the observed BMI distribution, with substantially greater uncertainty at higher BMI values.

### 3.2. Continuous Treatment Effects Modification by Baseline BMI

Table 2 presents the continuous treatment effect modification analyses for post-intervention anthropometric outcomes. The table also reports the adjusted IVR–control difference for each outcome at the overall mean baseline BMI (26.30 kg/m^2^), corresponding to the treatment coefficient from each model because baseline BMI was mean-centered. For the principal outcome, no statistically significant linear treatment-by-baseline BMI interaction was detected for post-intervention BMI (B = −0.039, 95% CI: −0.098 to 0.020; *p* = 0.189). The negative point estimate indicates that the estimated IVR–control difference became progressively more negative with increasing baseline BMI; however, the 95% CI included the null value, and the modest sample size and limited representation at the upper end of the BMI distribution preclude excluding potentially meaningful treatment effect heterogeneity. No statistically significant interactions were detected for body weight (B = −0.088, 95% CI: −0.254 to 0.077; Holm-adjusted *p* = 0.866), waist circumference (B = −0.029, 95% CI: −0.176 to 0.119; Holm-adjusted *p* = 0.866), or hip circumference (B = −0.129, 95% CI: −0.392 to 0.133; Holm-adjusted *p* = 0.866). Complete coefficient estimates for all continuous treatment effect modification models are provided in Supplementary Table S1.

To illustrate the magnitude and precision of the model-estimated treatment effect across the observed baseline BMI distribution, conditional IVR–control differences in post-intervention BMI were estimated at selected baseline BMI values. The estimated differences were −0.092 kg/m^2^ (95% CI: −0.453 to 0.270) at a baseline BMI of 22 kg/m^2^, −0.286 kg/m^2^ (95% CI: −0.553 to −0.020) at 27 kg/m^2^, −0.481 kg/m^2^ (95% CI: −0.909 to −0.053) at 32 kg/m^2^, and −0.676 kg/m^2^ (95% CI: −1.360 to 0.008) at 37 kg/m^2^. These pointwise estimates illustrate the direction and increasing uncertainty of the model-estimated treatment effect across the BMI distribution and should not be interpreted as separate evidence of statistically significant effect modification, for which the overall treatment × baseline BMI interaction remained the relevant test (*p* = 0.189).

**Figure 2:**
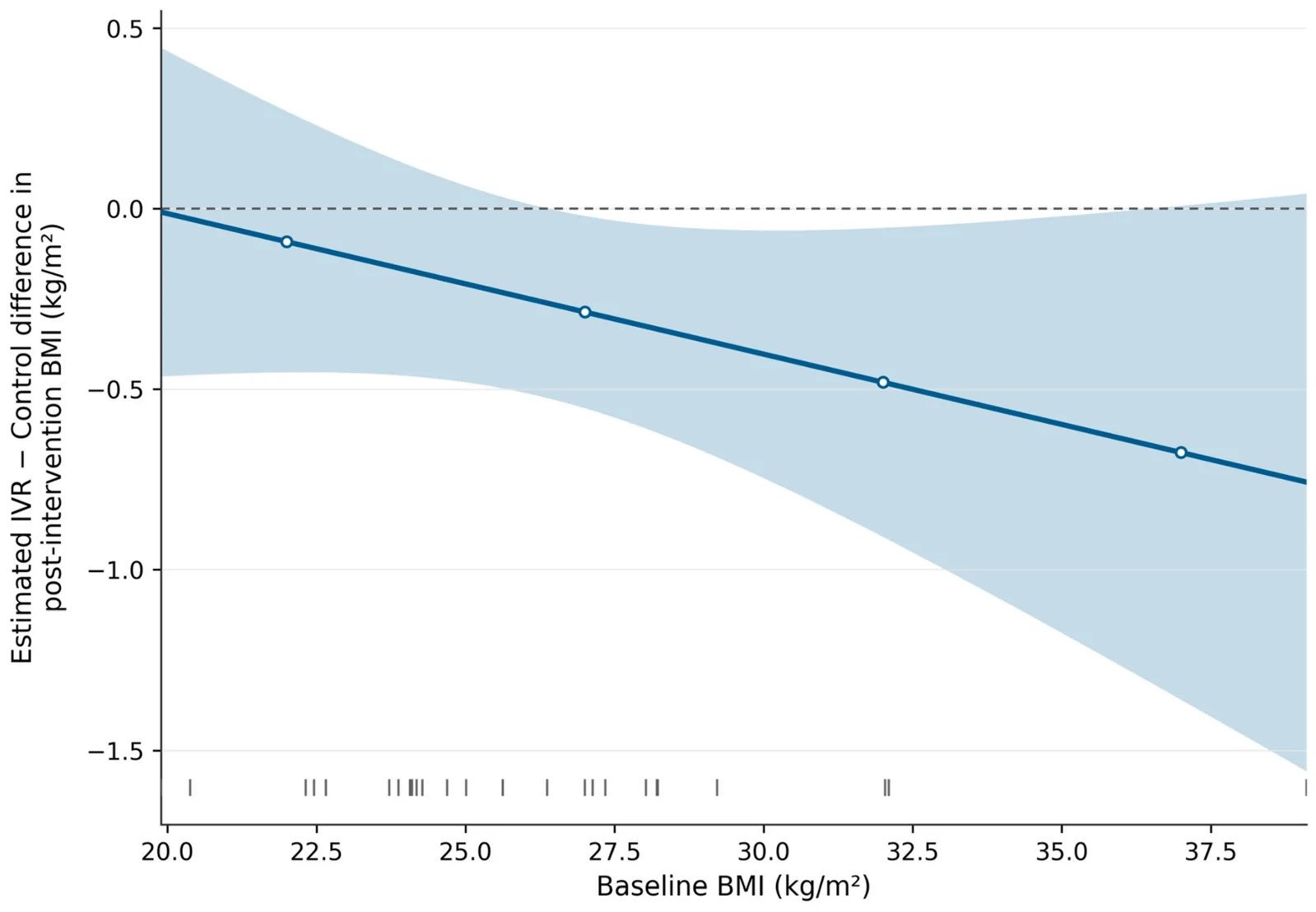
Model-estimated IVR–control difference in post-intervention BMI across the observed range of baseline BMI. The solid line represents the model-estimated IVR minus Control difference in post-intervention BMI, and the shaded area represents the 95% confidence interval. The horizontal dashed line indicates no estimated between-group difference. Rug marks represent the observed distribution of baseline BMI among the 60 participants. Point markers indicate baseline BMI values of 22, 27, 32, and 37 kg/m^2^, selected to illustrate conditional model estimates. Estimates were derived from the continuous treatment-by-baseline BMI interaction model. This analysis was exploratory, and no statistically significant linear treatment-by-baseline BMI interaction was detected.

Figure 2 illustrates the model-estimated IVR–control difference in post-intervention BMI across the observed range of baseline BMI. The confidence interval widened toward the extremes of the observed distribution, particularly at higher BMI values where fewer participants contributed information. Accordingly, although the point estimate became increasingly negative with increasing baseline BMI, uncertainty surrounding the estimated treatment effect increased substantially toward the upper end of the BMI distribution. Corresponding model-estimated treatment effect curves for BMI and the three secondary anthropometric outcomes are presented in Supplementary Figure S1.

### 3.3. Sensitivity and Exploratory Analyses

Adjustment for sex produced minimal changes in the estimated treatment-by-baseline BMI interactions. For post-intervention BMI, the sex-adjusted interaction estimate was B = −0.039 (95% CI: −0.099 to 0.022; *p* = 0.206), compared with B = −0.039 (95% CI: −0.098 to 0.020; *p* = 0.189) in the principal model. The corresponding interaction estimates for body weight, waist circumference, and hip circumference also remained statistically non-significant after adjustment for sex (Table S2).

Model diagnostics indicated no substantial multicollinearity (VIF range, 1.01–2.13) and no statistically significant evidence of heteroscedasticity according to the Breusch–Pagan tests (all *p* > 0.05). Shapiro–Wilk tests indicated departures from residual normality for all four models. Using the conventional Cook’s distance criterion of 4/N (0.067), six observations exceeded the threshold in the BMI model, four in the body-weight model, two in the waist-circumference model, and four in the hip-circumference model (Table S3). HC3 robust standard errors did not materially alter inference for any of the continuous interaction models (Table S4).

Across the four outcome-specific models, six unique participants exceeded the Cook’s distance threshold. Four had baseline BMI values of 39.10 kg/m^2^ and two had baseline BMI values of 27.13 kg/m^2^, indicating that influential observations were concentrated toward the upper end of the observed baseline BMI distribution. For the principal BMI outcome, exclusion of the six influential observations yielded an interaction estimate of B = −0.074 (95% CI: −0.154 to 0.007; *p* = 0.071; n = 54), compared with B = −0.039 (95% CI: −0.098 to 0.020; *p* = 0.189; n = 60) in the full-sample model. Secondary-outcome estimates were less stable after outcome-specific exclusion of influential observations: body weight, B = −0.156 (95% CI: −0.296 to −0.015; *p* = 0.031; n = 56); waist circumference, B = −0.012 (95% CI: −0.124 to 0.100; *p* = 0.828; n = 58); and hip circumference, B = −0.326 (95% CI: −0.589 to −0.064; *p* = 0.016; n = 56). These sensitivity findings were interpreted as indicating instability in some secondary-outcome estimates rather than as confirmatory evidence of treatment effect modification (Table S4).

Exploratory assessment of quadratic non-linearity did not indicate improved model fit for BMI, body weight, or waist circumference. For hip circumference, the quadratic specification showed evidence of improved fit relative to the corresponding linear model (nested F = 3.745, *p* = 0.030), with AIC decreasing from 271.21 to 267.28 and adjusted R^2^ increasing from 0.8892 to 0.8993. Given the modest sample size, the secondary nature of this outcome, and the exploratory model specification, this finding was considered hypothesis-generating and did not alter the principal linear analytical approach (Table S5).

Additional post hoc analyses categorizing baseline BMI as <25 versus ≥25 kg/m^2^ yielded interaction estimates of B = −0.678 (95% CI: −1.191 to −0.164; *p* = 0.011) for BMI, B = −1.711 (95% CI: −3.132 to −0.290; *p* = 0.020) for body weight, B = −0.729 (95% CI: −2.027 to 0.569; *p* = 0.265) for waist circumference, and B = −2.161 (95% CI: −4.309 to −0.013; *p* = 0.050) for hip circumference. Because this categorization was post hoc, involved dichotomization of a continuous effect modifier, and yielded results that differed from the principal continuous interaction analyses, these findings were considered hypothesis-generating and were not interpreted as confirmatory evidence of categorical treatment effect modification (Table S6).

Finally, the multiple-imputation sensitivity analysis including all 64 randomized participants yielded an interaction estimate nearly identical to that obtained in the principal complete-case analysis (B = −0.039, 95% CI: −0.097 to 0.020; *p* = 0.192). The corresponding complete-case estimate was B = −0.039 (95% CI: −0.098 to 0.020; *p* = 0.189). Thus, under the Missing At Random assumption used for the imputation analysis, the statistical interpretation of the treatment-by-baseline BMI interaction was not materially affected by the handling of the four missing post-intervention BMI values.

## 4. Discussion

In this exploratory secondary analysis of a randomized controlled trial, no statistically significant linear treatment-by-baseline BMI interaction was detected for post-intervention BMI or the secondary anthropometric outcomes of body weight, waist circumference, and hip circumference. For the principal outcome, the interaction estimate was negative, indicating that the model-estimated IVR–control difference in post-intervention BMI became progressively more negative with increasing baseline BMI; however, the 95% confidence interval included the null value. Importantly, the modest sample size, limited representation at the upper end of the baseline BMI distribution, and increasing uncertainty in the conditional estimates at higher BMI values preclude excluding potentially meaningful treatment effect heterogeneity. Additional post hoc analyses using a categorical BMI classification yielded different patterns from the principal continuous moderation analysis and were therefore considered hypothesis-generating rather than confirmatory. Taken together, these findings indicate that no statistically detectable linear effect modification by baseline BMI was identified in the present sample, rather than providing evidence of equivalent anthropometric responses across baseline BMI levels.

Previous studies have shown that IVR-based exercise interventions may influence anthropometric or body-composition outcomes in some populations, although findings have varied across studies [7,9]. However, these studies have primarily evaluated average intervention effects rather than whether individual baseline characteristics modify the magnitude of the response. In the parent randomized controlled trial from which the present data were derived, the IVR intervention improved cardiorespiratory fitness but did not produce statistically significant between-group effects on anthropometric outcomes [14]. The present exploratory analysis extends those findings by examining baseline BMI as a continuous potential treatment effect modifier. Although the direction of the estimated interaction suggested increasingly negative IVR–control differences at higher baseline BMI values, the interaction was not statistically significant and its precision was insufficient to establish BMI-dependent heterogeneity in anthropometric response.

The direction of the continuous interaction estimate warrants cautious consideration. Model-estimated IVR–control differences in post-intervention BMI became progressively more negative as baseline BMI increased, with conditional estimates of −0.092, −0.286, −0.481, and −0.676 kg/m^2^ at baseline BMI values of 22, 27, 32, and 37 kg/m^2^, respectively. However, these conditional estimates were derived from the same continuous interaction model and should not be interpreted as independent subgroup effects; inference regarding treatment effect modification should primarily rely on the treatment-by-baseline BMI interaction test [19]. Importantly, the confidence intervals widened toward the upper end of the baseline BMI distribution, where fewer participants contributed information, and the overall treatment-by-baseline BMI interaction remained statistically non-significant. Thus, although the direction of the point estimates may suggest a potential gradient in treatment response, the present data do not provide sufficiently precise evidence to establish such a pattern. This limitation is particularly relevant to the interpretation of participants with obesity: only eight participants had baseline BMI ≥30 kg/m^2^, and only two of these were allocated to the IVR group. Consequently, the present study cannot determine whether individuals with obesity exhibit a differential anthropometric response to the intervention.

The findings from the post hoc categorical analyses further illustrate the importance of how baseline BMI is modeled when evaluating potential treatment effect modification. When baseline BMI was dichotomized at 25 kg/m^2^, statistically significant treatment-by-BMI category interactions were observed for post-intervention BMI and body weight, whereas the corresponding continuous linear interaction analyses were not statistically significant. Categorization of a continuous variable can result in loss of information and statistical efficiency and may produce findings that depend on the selected cut-off point [15]. Moreover, the categorical analyses in the present study were post hoc, involved multiple anthropometric outcomes, and were conducted in a modest sample, further limiting the credibility of isolated nominally significant findings. Accordingly, the categorical results should not be interpreted as evidence that participants with BMI ≥25 kg/m^2^ experienced a differential intervention effect. Rather, the discrepancy between the continuous and categorical analyses highlights the uncertainty surrounding the form of any potential BMI-related treatment effect heterogeneity and supports treating these findings as hypothesis-generating.

From a practical perspective, the present findings are insufficient to determine whether baseline BMI is useful for identifying differential anthropometric responses to IVR-based exercise. Future trials specifically designed and adequately powered to investigate treatment effect heterogeneity should evaluate baseline BMI alongside other plausible biological and behavioral modifiers, including sex, baseline physical fitness, habitual physical activity, and intervention adherence.

Several sensitivity analyses were conducted to examine the robustness of the interaction estimates under alternative model specifications and assumptions. Adjustment for sex, which was relevant given the baseline imbalance in sex distribution between the randomized groups, produced minimal change in the treatment-by-baseline BMI interaction for the principal outcome. Similarly, the use of HC3 heteroscedasticity-consistent standard errors did not materially alter the statistical interpretation of the continuous interaction models. The principal BMI interaction also remained statistically non-significant after exclusion of observations exceeding the prespecified Cook’s distance threshold, although the estimate became more negative and approached conventional statistical significance. In contrast, the interaction estimates for body weight and hip circumference became nominally statistically significant after exclusion of influential observations, indicating that these secondary findings were sensitive to a small number of participants and should not be considered stable evidence of treatment effect modification. Influential observations were concentrated toward the upper end of the baseline BMI distribution, further emphasizing the limited information available for estimating treatment effects at higher BMI values. Exploratory quadratic models provided no evidence of improved model fit for BMI, body weight, or waist circumference, whereas a quadratic specification improved model fit for hip circumference; given the small sample and exploratory nature of this analysis, this finding should be considered hypothesis-generating. Finally, multiple imputation including all 64 randomized participants yielded a principal BMI interaction estimate nearly identical to the complete-case estimate, indicating that the statistical interpretation of this interaction was not materially changed under the Missing at Random assumption used for the imputation analysis.

This study has several strengths. It used data from a randomized controlled trial with standardized anthropometric assessments and examined treatment effect modification using baseline BMI primarily as a continuous variable. The regression models incorporated the corresponding baseline outcome and formally tested treatment-by-baseline BMI interactions rather than inferring differential responses from separate within-group or subgroup comparisons. The revised analytical framework also included sex-adjusted models, heteroscedasticity-consistent standard errors, influence diagnostics, exploratory assessment of nonlinearity, and multiple imputation including all randomized participants. Importantly, the sensitivity analyses were interpreted according to their consistency with the principal analysis rather than being used selectively to support a particular conclusion. The availability of the parent randomized trial report [14], together with the reproducible analytical workflow provided as Supplementary Code S1, further enhances transparency and allows the present exploratory moderation analyses to be distinguished from the prespecified objectives and previously reported findings of the parent trial.

Several limitations should be considered when interpreting these findings. First, the treatment effect modification analyses were exploratory and were not prespecified in the parent trial protocol or registry, and the parent trial was designed to evaluate average intervention effects rather than being specifically powered to detect treatment-by-baseline BMI interactions. Consequently, the absence of statistically significant linear interactions should not be interpreted as evidence of homogeneous treatment effects, and potentially meaningful heterogeneity cannot be excluded. Second, the modest sample size and sparse representation at the upper end of the BMI distribution, particularly the small number of participants with obesity, limited the precision of effect estimates at higher baseline BMI values. The imbalance in sex distribution between randomized groups represents an additional consideration, although adjustment for sex produced minimal change in the principal interaction estimate. Third, the principal analyses were based on the 60 participants with post-intervention anthropometric data rather than all 64 randomized participants. The four participants without post-intervention data had completed baseline assessments, and the two allocated to IVR had partial intervention exposure before discontinuation. Although multiple imputation including all randomized participants yielded a principal interaction estimate nearly identical to the complete-case result under the Missing At Random assumption, attrition bias cannot be excluded. Fourth, influence diagnostics identified a small number of observations concentrated toward the upper end of the baseline BMI distribution, and exclusion of influential observations altered some secondary interaction estimates, particularly for body weight and hip circumference, underscoring the limited stability of these secondary findings. In addition, multiple exploratory model specifications and sensitivity analyses were examined, including sex-adjusted, heteroscedasticity-consistent, influence-restricted, nonlinear, categorical, and multiple-imputation analyses. Although these analyses were used to assess robustness and model assumptions, their number increases the potential for chance findings and reinforces the hypothesis-generating nature of the results. Fifth, although exercise intensity was monitored in real time during the intervention, session-level heart-rate data were not retained, preventing retrospective quantification of the intensity achieved across sessions. Although adherence could be characterized from session attendance, attendance alone does not quantify the exercise dose completed within each session, and detailed session-level adherence to the prescribed intensity was unavailable. Therefore, the prescribed exercise intensity cannot be assumed to represent the actual internal exercise dose achieved by individual participants, and interindividual variability in the training stimulus may have contributed to heterogeneity in anthropometric responses. Dietary intake and physical activity or exercise performed outside the intervention were also not formally monitored or controlled; therefore, unmeasured behavioral changes may have influenced both the average anthropometric changes observed during the trial and interindividual variability in anthropometric response. Finally, although anthropometric assessments followed standardized procedures and were performed by trained evaluators, study-specific technical error of measurement or test–retest reliability estimates were not available. Given the relatively small anthropometric changes observed, particularly for circumference measurements, and the absence of study-specific technical error estimates, it cannot be determined whether some changes exceeded the expected measurement variability of the anthropometric procedures. These small changes should therefore be interpreted cautiously rather than assumed to represent true biological change.

## 5. Conclusions

In this exploratory secondary analysis of a randomized controlled trial, no statistically significant linear treatment-by-baseline BMI interaction was detected for post-intervention BMI or the secondary anthropometric outcomes of body weight, waist circumference, and hip circumference. However, the modest sample size, the fact that the parent trial was not specifically designed or powered to evaluate treatment effect modification, limited representation of participants at the upper end of the BMI distribution, and widening uncertainty in the conditional estimates preclude excluding potentially meaningful treatment effect heterogeneity. Post hoc categorical analyses yielded different patterns from the principal continuous moderation analysis and should therefore be considered hypothesis-generating rather than confirmatory. Moreover, some secondary interaction estimates changed materially in influence-based sensitivity analyses, further limiting the stability of these secondary findings. Accordingly, the present findings should not be interpreted as evidence of equivalent anthropometric responses across baseline BMI levels. Future randomized controlled trials specifically designed and adequately powered to evaluate treatment effect modification, with sufficient representation across the BMI distribution, are needed to determine whether baseline BMI or other participant characteristics influence anthropometric responses to IVR-based exercise interventions.

## Figures and Tables

**Table 1:** Baseline characteristics of participants included in the principal complete-case analysis according to randomized treatment allocation.

Variable	IVR (n = 30)	Control (n = 30)	Standardized Difference
Female, n (%)	8 (26.7)	14 (46.7)	−0.42
Age (years)	21.38 ± 1.22	21.78 ± 1.33	−0.31
Height (cm)	170.86 ± 6.70	169.64 ± 9.64	0.15
Body weight (kg)	76.39 ± 16.87	77.37 ± 19.52	−0.05
BMI (kg/m^2^)	25.95 ± 4.18	26.65 ± 5.01	−0.15
Waist circumference (cm)	82.29 ± 11.45	81.40 ± 13.52	0.07
Hip circumference (cm)	100.72 ± 7.86	100.36 ± 7.65	0.05
**BMI category, n (%)**			
<25 kg/m^2^	14 (46.7)	14 (46.7)	—
25–29.9 kg/m^2^	14 (46.7)	10 (33.3)	—
≥30 kg/m^2^	2 (6.7)	6 (20.0)	—

Data are presented as mean ± SD unless otherwise indicated. The table includes the 60 participants with both baseline and post-intervention anthropometric data who were included in the principal complete-case analysis. Standardized differences are expressed as Cohen’s d for continuous variables. For sex, the standardized difference in proportions was calculated as (pIVR − pControl)/√{[pIVR(1 − pIVR) + pControl(1 − pControl)]/2}, where pIVR and pControl represent the proportions of female participants in the IVR and control groups, respectively. Positive values indicate higher values in the IVR group. Baseline hypothesis tests were not performed because between-group significance testing of baseline characteristics is not informative for assessing the success of randomization. BMI, body mass index; IVR, immersive virtual reality; SD, standard deviation.

**Table 2:** Continuous treatment effect modification by baseline body mass index for post-intervention anthropometric outcomes.

Outcome	Adjusted IVR-Control Difference, B (95% CI)	Interaction B	SE	95% CI	*p*-Value	Holm-Adjusted *p* †
**Principal outcome**						
BMI (kg/m^2^)	−0.259 (−0.522 to 0.004)	−0.039	0.029	−0.098 to 0.020	0.189	—
**Secondary outcomes**						
Body weight (kg)	−0.664 (−1.404 to 0.076)	−0.088	0.083	−0.254 to 0.077	0.289	0.866
Waist circumference (cm)	−0.139 (−0.806 to 0.527)	−0.029	0.074	−0.176 to 0.119	0.700	0.866
Hip circumference (cm)	−0.160 (−1.331 to 1.011)	−0.129	0.131	−0.392 to 0.133	0.326	0.866

The adjusted IVR–control difference represents the treatment coefficient from each model and corresponds to the estimated between-group difference at the overall mean baseline BMI (26.30 kg/m^2^), because baseline BMI was mean-centered. Interaction B represents the coefficient for the treatment × baseline BMI interaction from ordinary least-squares regression models. Treatment allocation was coded as 0 (control) and 1 (IVR), and baseline BMI was centered at the overall sample mean (26.30 kg/m^2^). For post-intervention BMI, the model included treatment allocation, centered baseline BMI, and their interaction. Models for body weight, waist circumference, and hip circumference additionally included the corresponding baseline value of each outcome as a covariate. Negative interaction coefficients indicate an increasingly negative estimated IVR–control difference with increasing baseline BMI. All interaction analyses were exploratory and were not prespecified in the parent trial protocol or registry. † Holm-adjusted *p*-values apply only to the three secondary interaction tests. BMI, body mass index; CI, confidence interval; IVR, immersive virtual reality; SE, standard error.

## Data Availability

The data presented in this study are available on request from the corresponding author. The data are not publicly available due to ethical standards.

## Supplementary Materials

The following supporting information can be downloaded at: https://www.mdpi.com/article/10.3390/healthcare14183073/s1, Table S1: Complete regression models evaluating continuous treatment effect modification by baseline body mass index for post-intervention anthropometric outcomes; Table S2: Sensitivity analyses of continuous treatment effect modification models additionally adjusted for sex; Table S3: Diagnostic assessment of the continuous treatment effect modification models; Table S4: Sensitivity analyses of continuous treatment effect modification using HC3 robust standard errors and exclusion of influential observations; Table S5: Exploratory assessment of quadratic non-linearity in treatment effect modification by baseline body mass index; Table S6: Post hoc exploratory categorical treatment effect modification analyses according to baseline body mass index; Figure S1: Model-estimated IVR–control differences across the observed range of baseline body mass index for anthropometric outcomes; Supplementary Code S1: Reproducible Python code for the exploratory secondary analysis of treatment effect modification by baseline body mass index.

## Abbreviations

The following abbreviations are used in this manuscript:

ANCOVA	Analysis of Covariance
BMI	Body Mass Index
CI	Confidence Interval
IVR	Immersive Virtual Reality
SD	Standard deviation
SE	Standard error
VIF	Variance Inflation Factor

## References

[1] GBD 2021 Adult BMI Collaborators (2025). Global, regional, and national prevalence of adult overweight and obesity, 1990–2021, with forecasts to 2050: A forecasting study for the Global Burden of Disease Study 2021. Lancet.

[2] World Health Organization Obesity and Overweight. https://www.who.int/news-room/fact-sheets/detail/obesity-and-overweight.

[3] World Health Organization Physical Activity. https://www.who.int/news-room/fact-sheets/detail/physical-activity.

[4] Baillot A., Chenail S., Polita N.B., Simoneau M., Libourel M., Nazon E., Riesco E., Bond D.S., Romain A.J. (2021). Physical activity motives, barriers, and preferences in people with obesity: A systematic review. PLoS ONE.

[5] Hamer O., Larkin D., Relph N., Dey P. (2021). Fear-related barriers to physical activity among adults with overweight and obesity: A narrative synthesis scoping review. Obes. Rev..

[6] Rubino F., Puhl R.M., Cummings D.E., Eckel R.H., Ryan D.H., Mechanick J.I., Nadglowski J., Ramos Salas X., Schauer P.R., Twenefour D. (2020). Joint international consensus statement for ending stigma of obesity. Nat. Med..

[7] Mocco A., Valmaggia L., Bernardi L., Alfieri M., Tarricone I. (2024). Enhancing physical activity with immersive virtual reality: A systematic review. Cyberpsychol. Behav. Soc. Netw..

[8] Giakoni-Ramírez F., Godoy-Cumillaf A., Espoz-Lazo S., Duclos-Bastias D., Del Val Martín P. (2023). Physical activity in immersive virtual reality: A scoping review. Healthcare.

[9] Brown R.C.C., Ross M.H., Russell T.G. (2026). Clinical effectiveness of immersive virtual reality exercise interventions: Systematic review and meta-analysis of randomized controlled trials. J. Med. Internet Res..

[10] McGhee W.R.G., Doherty C.J., Graham-Wisener L., Fallis R., Stone C., Axiaq A., Dempster M. (2024). Immersive virtual reality and psychological well-being in adult chronic physical illness: Systematic review. BMJ Support. Palliat. Care.

[11] Giakoni-Ramírez F., Godoy-Cumillaf A., Fuentes-Merino P., Farías-Valenzuela C., Duclos-Bastías D., Bruneau-Chávez J., Merellano-Navarro E., Velásquez-Olavarría R. (2023). Intensity of a physical exercise programme executed through immersive virtual reality. Healthcare.

[12] Wang R., Lagakos S.W., Ware J.H., Hunter D.J., Drazen J.M. (2007). Statistics in medicine—Reporting of subgroup analyses in clinical trials. N. Engl. J. Med..

[13] Alosh M., Huque M.F., Bretz F., D’Agostino R.B. (2017). Tutorial on statistical considerations on subgroup analysis in confirmatory clinical trials. Stat. Med..

[14] Godoy-Cumillaf A., Fuentes-Merino P., de Souza-Lima J., Giakoni-Ramírez F., Muñoz-Strale C., Parra-Saldias M., Duclos-Bastias D., Farias-Valenzuela C., Merellano-Navarro E., Bruneau-Chávez J. (2026). The effect of an immersive virtual reality physical activity intervention on anthropometric variables, physical fitness, and blood pressure in college students: A randomized controlled trial. Healthcare.

[15] Hopewell S., Chan A.W., Collins G.S., Hróbjartsson A., Moher D., Schulz K.F., Tunn R., Aggarwal R., Berkwits M., Berlin J.A. (2025). CONSORT 2025 statement: Updated guideline for reporting randomised trials. BMJ.

[16] Fuentes-Merino P., Godoy-Cumillaf A., Giakoni-Ramírez F., Duclos-Bastías D., Bruneau-Chávez J., Merellano-Navarro E., Bizzozero-Peroni B. (2025). Effects of a physical exercise program executed through immersive virtual reality on physical fitness and body composition in college adults: Protocol for a randomized controlled trial. Retos.

[17] Vickers A.J., Altman D.G. (2001). Analysing controlled trials with baseline and follow up measurements. BMJ.

[18] Holm S. (1979). A simple sequentially rejective multiple test procedure. Scand. J. Stat..

[19] Sun X., Briel M., Walter S.D., Guyatt G.H. (2010). Is a subgroup effect believable? Updating criteria to evaluate the credibility of subgroup analyses. BMJ.

