# Supplementary material for: Baseline Body Mass Index and Anthropometric Responses to an Immersive Virtual Reality-Based Exercise Intervention: An Exploratory Secondary Analysis of a Randomized Controlled Trial
Source: Healthcare (Basel). 2026 Sep 18;14(18):3073. doi: 10.3390/healthcare14183073 (PMC13606850; doi:10.3390/healthcare14183073)
Supplement: Supplementary file 1 [file healthcare-14-03073-s001.zip › healthcare-4529740-supplementary.pdf]

Table S1. Complete continuous regression models evaluating treatment-effect modification by baseline body mass index for post-intervention anthropometric outcomes.

Outcome	Predictor	B	SE	95% CI	*p*-value	Adjusted R^2	N
**Panel A. Principal outcome**							
BMI (kg/m^2)	Treatment	−0.259	0.131	−0.522 to 0.004	0.053	0.986	60
	Baseline BMI (centered)	0.954	0.019	0.917 to 0.992	<0.001	0.986	60
	Treatment × Baseline BMI (centered)	−0.039	0.029	−0.098 to 0.020	0.189	0.986	60
**Panel B. Secondary outcomes**							
Body weight (kg)	Baseline body weight	0.915	0.027	0.862 to 0.969	<0.001	0.993	60
	Treatment	−0.664	0.369	−1.404 to 0.076	0.078	0.993	60
	Baseline BMI (centered)	0.147	0.107	−0.066 to 0.361	0.172	0.993	60
	Treatment × Baseline BMI (centered)	−0.088	0.083	−0.254 to 0.077	0.289	0.993	60
Waist circumference (cm)	Baseline waist circumference	0.956	0.026	0.904 to 1.007	<0.001	0.989	60
	Treatment	−0.139	0.333	−0.806 to 0.527	0.677	0.989	60
	Baseline BMI (centered)	0.044	0.072	−0.099 to 0.188	0.539	0.989	60
	Treatment × Baseline BMI (centered)	−0.029	0.074	−0.176 to 0.119	0.700	0.989	60
Hip circumference (cm)	Baseline hip circumference	0.816	0.067	0.682 to 0.951	<0.001	0.889	60
	Treatment	−0.160	0.584	−1.331 to 1.011	0.785	0.889	60
	Baseline BMI (centered)	0.075	0.117	−0.161 to 0.310	0.527	0.889	60
	Treatment × Baseline BMI (centered)	−0.129	0.131	−0.392 to 0.133	0.326	0.889	60

Ordinary least-squares regression estimates are presented for the complete continuous treatment-effect modification models. Treatment allocation was coded as 0 = control and 1 = immersive virtual reality (IVR). Baseline body mass index (BMI) was centered at the overall sample mean (26.30 kg/m^2). For post-intervention BMI, the model included treatment allocation, centered baseline BMI, and the treatment × baseline BMI interaction. For post-intervention body weight, waist circumference, and hip circumference, the corresponding baseline value of each outcome was additionally included as a covariate. Regression coefficients (B), standard errors (SE), 95% confidence intervals (95% CI), p-values, and adjusted coefficients of determination (adjusted R^2) are reported. The treatment × baseline BMI interaction represents the test of linear treatment-effect modification. All models included N = 60 participants in the principal complete-case analysis; residual degrees of freedom were 56 for the BMI model and 55 for the body weight, waist circumference, and hip circumference models. All treatment-effect modification analyses were exploratory and were not prespecified in the parent trial protocol or registry. The high adjusted R^2 values primarily reflect the strong association between post-intervention anthropometric outcomes and their corresponding baseline

measurements and should not be interpreted as evidence of strong explanatory performance attributable to the intervention or baseline BMI as a treatment-effect modifier. BMI, body mass index; CI, confidence interval; IVR, immersive virtual reality; SE, standard error.

Table S2. Sensitivity analyses of continuous treatment-effect modification models additionally adjusted for sex.

Outcome	Model	Interaction B	SE	95% CI	*p*-value
**Principal outcome**					
BMI (kg/m²)	Principal model	−0.039	0.029	−0.098 to 0.020	0.189
	Sex-adjusted model	−0.039	0.030	−0.099 to 0.022	0.206
**Secondary outcomes**					
Body weight (kg)	Principal model	−0.088	0.083	−0.254 to 0.077	0.289
	Sex-adjusted model	−0.082	0.085	−0.252 to 0.089	0.339
Waist circumference (cm)	Principal model	−0.029	0.074	−0.176 to 0.119	0.700
	Sex-adjusted model	−0.022	0.077	−0.175 to 0.131	0.774
Hip circumference (cm)	Principal model	−0.129	0.131	−0.392 to 0.133	0.326
	Sex-adjusted model	−0.053	0.126	−0.306 to 0.201	0.677

Values represent the treatment × baseline body mass index (BMI) interaction coefficient from the principal complete-case models and corresponding sensitivity models additionally adjusted for sex. Treatment allocation was coded as 0 = control and 1 = immersive virtual reality (IVR), and baseline BMI was centered at the overall sample mean (26.30 kg/m²). For post-intervention BMI, models included treatment allocation, centered baseline BMI, and their interaction; the sex-adjusted model additionally included sex as a covariate. Models for post-intervention body weight, waist circumference, and hip circumference additionally included the corresponding baseline value of each outcome. All models included N = 60 participants. The sex-adjusted analyses were conducted as sensitivity analyses to assess the potential influence of the baseline sex imbalance between randomized groups. All treatment-effect modification analyses were exploratory. B, regression coefficient; BMI, body mass index; CI, confidence interval; IVR, immersive virtual reality; SE, standard error.

Table S3. Diagnostic assessment of the continuous treatment-effect modification models.

Outcome	N	Residual df	Shapiro–Wilk *p*	Breusch–Pagan *p*	VIF range	Maximum Cook's distance	Cook's distance threshold	Cases exceeding threshold, n	Maximum leverage	∣Studentized residual∣ > 3, n
BMI	60	56	0.002	0.155	1.01–1.70	0.101	0.067	6	0.375	2
Body weight	60	55	<0.001	0.326	1.71–2.13	0.092	0.067	4	0.381	2
Waist circumference	60	55	<0.001	0.085	1.70–2.09	0.098	0.067	2	0.376	2
Hip circumference	60	55	<0.001	0.336	1.71–2.04	0.203	0.067	4	0.377	2

Diagnostic statistics are presented for the ordinary least-squares continuous treatment-effect modification models. Residual normality was assessed using the Shapiro–Wilk test and homoscedasticity using the Breusch–Pagan test. Multicollinearity was assessed using variance inflation factors (VIF), calculated for the predictor matrix without the intercept. Potentially influential observations were assessed using Cook's distance, with a conventional threshold of 4/N (0.067 for N = 60), together with leverage values and externally studentized residuals. The column "Cases exceeding threshold"

indicates the number of observations with Cook's distance >4/N, whereas the final column indicates the number of observations with an absolute externally studentized residual >3. BMI, body mass index; df, degrees of freedom; VIF, variance inflation factor.

Table S4. Sensitivity analyses of continuous treatment-effect modification using HC3 robust standard errors and exclusion of influential observations.

Outcome	Analysis	N	Interaction B	SE	95% CI	*p*-value
**Principal outcome**						
BMI (kg/m²)	Full-sample OLS	60	−0.039	0.029	−0.098 to 0.020	0.189
	Full-sample HC3	60	−0.039	0.025	−0.090 to 0.012	0.132
	Excluding Cook's distance >4/N	54	−0.074	0.040	−0.154 to 0.007	0.071
**Secondary outcomes**						
Body weight (kg)	Full-sample OLS	60	−0.088	0.083	−0.254 to 0.077	0.289
	Full-sample HC3	60	−0.088	0.064	−0.217 to 0.040	0.172
	Excluding Cook's distance >4/N	56	−0.156	0.070	−0.296 to −0.015	0.031
Waist circumference (cm)	Full-sample OLS	60	−0.029	0.074	−0.176 to 0.119	0.700
	Full-sample HC3	60	−0.029	0.040	−0.109 to 0.051	0.478
	Excluding Cook's distance >4/N	58	−0.012	0.056	−0.124 to 0.100	0.828
Hip circumference (cm)	Full-sample OLS	60	−0.129	0.131	−0.392 to 0.133	0.326
	Full-sample HC3	60	−0.129	0.151	−0.431 to 0.172	0.394
	Excluding Cook's distance >4/N	56	−0.326	0.131	−0.589 to −0.064	0.016

Values represent the treatment × baseline body mass index (BMI) interaction coefficient from sensitivity analyses of the continuous treatment-effect modification models. Full-sample ordinary least-squares (OLS) estimates are shown for reference and compared with estimates obtained using HC3 heteroscedasticity-consistent standard errors and with models refitted after excluding observations with Cook's distance >4/N. With N = 60 in the full analytical sample, the Cook's distance threshold was 0.067. The number of observations exceeding this threshold was six for BMI, four for body weight, two for waist circumference, and four for hip circumference, resulting in analytical samples of 54, 56, 58, and 56 participants, respectively, after exclusion. Treatment allocation was coded as 0 = control and 1 = immersive virtual reality (IVR), and baseline BMI was centered at the overall sample mean (26.30 kg/m²). For post-intervention BMI, models included treatment allocation, centered baseline BMI, and their interaction; models for body weight, waist circumference, and hip circumference additionally included the corresponding baseline value of each outcome. These analyses were conducted as sensitivity analyses and should not be interpreted as alternative primary models. Across the four outcome-specific models, six unique participants exceeded the Cook's distance threshold. Four of these participants had baseline BMI values of 39.10 kg/m² and two had baseline BMI values of 27.13 kg/m². The influential observations were therefore concentrated toward the upper end of the observed baseline BMI distribution. In the exploratory categorical analysis, 14 of 30 participants (46.7%) in the IVR group and 14 of 30 (46.7%) in the control group had baseline BMI <25 kg/m², whereas 16 of 30 participants (53.3%) in each group had baseline BMI ≥25 kg/m². BMI, body mass index; CI, confidence interval; HC3, heteroscedasticity-consistent covariance estimator; IVR, immersive virtual reality; OLS, ordinary least squares; SE, standard error.

Table S5. Exploratory assessment of quadratic non-linearity in treatment-effect modification by baseline body mass index.

Outcome	Linear model AIC	Quadratic model AIC	Linear model adjusted R^2	Quadratic model adjusted R^2	Nested F statistic	df difference	Nested F-test p-value
BMI (kg/m^2)	92.44	92.56	0.9864	0.9868	1.803	2	0.1745
Body weight (kg)	216.83	218.66	0.9931	0.9931	0.973	2	0.3847
Waist circumference (cm)	202.68	206.63	0.9891	0.9887	0.022	2	0.9781
Hip circumference (cm)	271.21	267.28	0.8892	0.8993	3.745	2	0.0301

Quadratic models were fitted as exploratory sensitivity analyses to assess potential departures from the linear treatment-effect modification specification. Each quadratic model extended the corresponding continuous linear model by including a squared centered baseline BMI term and its interaction with treatment allocation (baseline BMI^2 and treatment × baseline BMI^2). For body weight, waist circumference, and hip circumference, the corresponding baseline value of each outcome was additionally included as a covariate in both the linear and quadratic models. Baseline BMI was centered at the overall sample mean (26.30 kg/m^2), and all models included N = 60 participants. Nested F-tests were used to compare each quadratic model with its corresponding linear model. Given the modest analytical sample, the exploratory nature of these analyses, and the additional model complexity, quadratic models were not used as the principal analytical specification. Any indication of improved nonlinear fit was considered hypothesis-generating and was not interpreted as confirmatory evidence of nonlinear treatment-effect modification. AIC, Akaike information criterion; BMI, body mass index; df, degrees of freedom.

Table S6. Post hoc exploratory categorical treatment-effect modification analyses according to baseline body mass index.

Outcome	Interaction B	SE	95% CI	p-value
BMI (kg/m^2)	−0.678	0.256	−1.191 to −0.164	0.011
Body weight (kg)	−1.711	0.709	−3.132 to −0.290	0.020
Waist circumference (cm)	−0.729	0.648	−2.027 to 0.569	0.265
Hip circumference (cm)	−2.161	1.072	−4.309 to −0.013	0.050

Values represent the treatment × baseline BMI category interaction coefficient from exploratory ordinary least-squares regression models. Baseline BMI was categorized post hoc as <25 kg/m^2 versus ≥25 kg/m^2, with BMI <25 kg/m^2 as the reference category. Treatment allocation was coded as 0 = control and 1 = immersive virtual reality (IVR). For post-intervention BMI, the model included baseline BMI as a continuous covariate, treatment allocation, baseline BMI category, and the treatment × baseline BMI category interaction. Models for post-intervention body weight, waist circumference, and hip circumference included the corresponding baseline value of each outcome, treatment allocation, baseline BMI category, and their interaction. Among the 60 participants included in the complete-case analysis, 28 had baseline BMI <25 kg/m^2 (14 IVR and 14 control) and 32 had baseline BMI ≥25 kg/m^2 (16 IVR and 16 control). These categorical analyses were not prespecified in the parent

trial protocol or registry and were conducted as additional post hoc exploratory analyses. Because categorizing a continuous variable reduces information and may produce results that differ from the continuous interaction analysis, these estimates should be interpreted as hypothesis-generating and not as confirmatory evidence of treatment-effect modification. No multiplicity adjustment was applied to these post hoc categorical analyses. BMI, body mass index; CI, confidence interval; IVR, immersive virtual reality; SE, standard error.

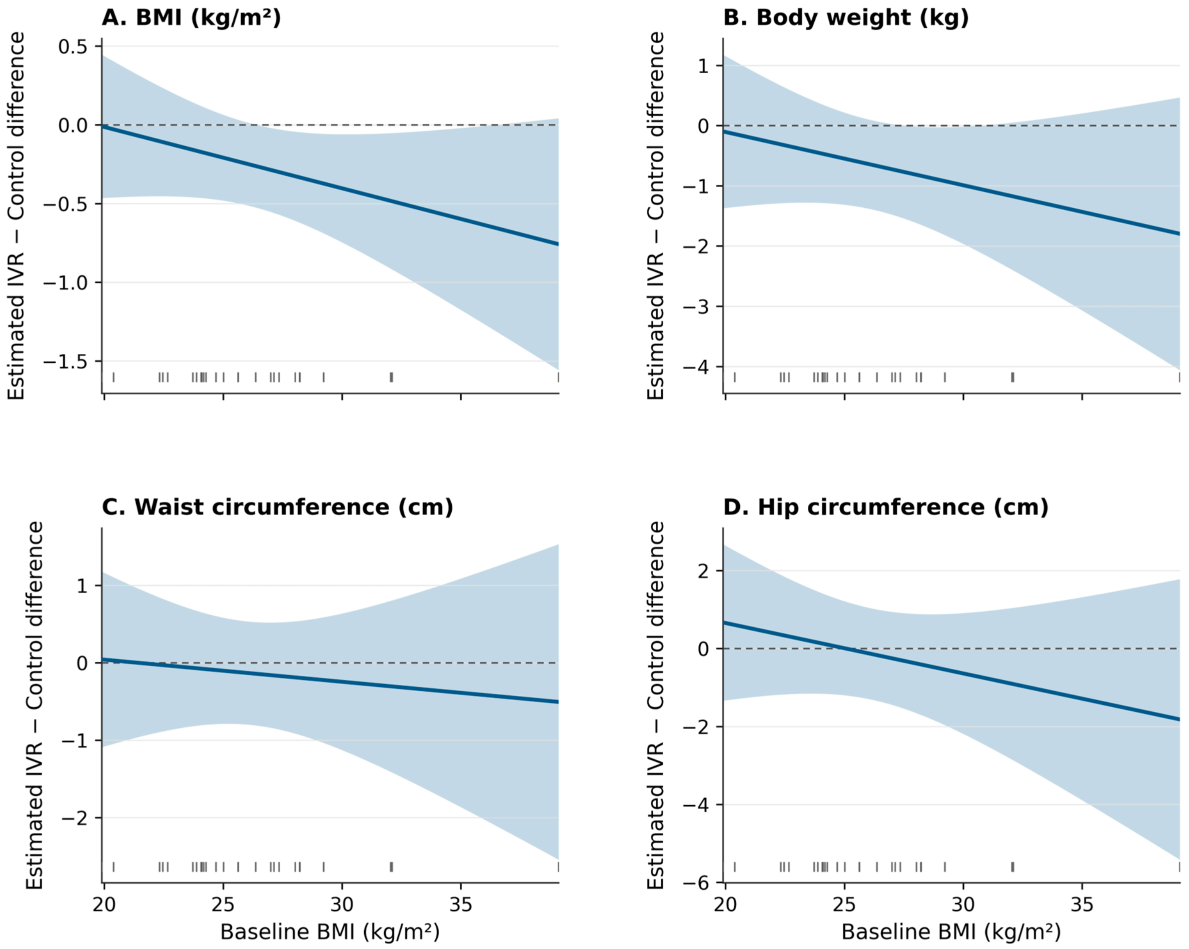

Figure S1. Model-estimated IVR–control differences across the observed range of baseline body mass index for anthropometric outcomes. Panels show model-estimated IVR minus Control differences in post-intervention BMI (A), body weight (B), waist circumference (C), and hip circumference (D) across the observed range of baseline BMI. Solid lines represent estimated treatment effects, shaded areas represent 95% confidence intervals, horizontal dashed lines indicate no estimated between-group difference, and rug marks represent the observed distribution of baseline BMI among the 60 participants included in the principal complete-case analysis. Estimates were derived from the respective continuous linear treatment-by-baseline BMI interaction models. These treatment-effect modification analyses were exploratory and were not prespecified in the parent trial protocol or registry. BMI, body mass index; IVR, immersive virtual reality.

Supplementary Code S1. Reproducible Python code for the exploratory secondary analysis of treatment-effect modification by baseline body mass index.

Input files expected in the working directory:

```
    2RCT_IVR.xlsx

    Base_64_randomized_MI_sensitivity.xlsx

"""


import sys

from pathlib import Path


import numpy as np

import pandas as pd

import scipy

from scipy import stats

import statsmodels

import statsmodels.api as sm

import statsmodels.formula.api as smf

from statsmodels.stats.anova import anova_lm

from statsmodels.stats.diagnostic import het_breuschpagan

from statsmodels.stats.multitest import multipletests

from statsmodels.stats.outliers_influence import variance_inflation_factor

import matplotlib.pyplot as plt



# ============================================================================

# 1. SOFTWARE, PATHS, AND HELPER FUNCTIONS

# ============================================================================


COMPLETE_CASE_FILE = Path("2RCT_IVR.xlsx")

RANDOMIZED_FILE = Path("Base_64_randomized_MI_sensitivity.xlsx")

RANDOMIZED_SHEET = "Data_64"
```

```
OUTPUT_DIR = Path("Supplementary_Code_S1_outputs")
OUTPUT_DIR.mkdir(exist_ok=True)

RANDOM_SEED = 20260902
N_IMPUTATIONS = 50

print("SUPPLEMENTARY CODE S1")
print("Runtime software versions")
print(f"Python: {sys.version}")
print(f"pandas: {pd.__version__}")
print(f"NumPy: {np.__version__}")
print(f"SciPy: {scipy.__version__}")
print(f"statsmodels: {statsmodels.__version__}")
print()


def p_text(value):
    """Format p-values for compact printed output."""
    return "<0.001" if value < 0.001 else f"{value:.6f}"


def model_coefficient_table(model, outcome_name):
    """Return conventional OLS coefficient results for one model."""
    confidence_intervals = model.conf_int()
    results = pd.DataFrame(
        {
            "Outcome": outcome_name,
            "Term": model.params.index,
```

```
            "B": model.params.values,
            "SE": model.bse.values,
            "95% CI lower": confidence_intervals.iloc[:, 0].values,
            "95% CI upper": confidence_intervals.iloc[:, 1].values,
            "p-value": model.pvalues.values,
        }
    )
    results["Adjusted R²"] = model.rsquared_adj
    results["N"] = int(model.nobs)
    results["Residual df"] = int(model.df_resid)
    return results


def interaction_result(model, interaction_term):
    """Extract interaction coefficient results from a fitted OLS model."""
    confidence_intervals = model.conf_int().loc[interaction_term]
    return {
        "Interaction B": model.params[interaction_term],
        "SE": model.bse[interaction_term],
        "95% CI lower": confidence_intervals.iloc[0],
        "95% CI upper": confidence_intervals.iloc[1],
        "p-value": model.pvalues[interaction_term],
    }


def check_close(name, observed, expected, tolerance, verification_messages):
    """Store an internal numerical verification result without altering models."""
    passed = bool(np.isclose(observed, expected, rtol=0, atol=tolerance))
```

```
    status = "PASS" if passed else "VERIFICATION FAILED"
    verification_messages.append(
        {
            "Check": name,
            "Status": status,
            "Observed": observed,
            "Expected": expected,
            "Tolerance": tolerance,
        }
    )
    return passed


# ============================================================================
# 2. DATA PREPARATION: N=60 COMPLETE-CASE DATASET
# ============================================================================

cc_data = pd.read_excel(COMPLETE_CASE_FILE, sheet_name="Hoja1")
cc_data.columns = [str(column).strip() for column in cc_data.columns]

rename_columns = {
    "Nombre": "ID",
    "Género": "Sex",
    "Talla1": "Height_baseline",
    "Talla2": "Height_post",
    "Peso1": "Weight_baseline",
    "Peso2": "Weight_post",
    "IMC1": "BMI_baseline",
```

```
    "IMC2": "BMI_post",
    "Cintura1": "Waist_baseline",
    "Cintura2": "Waist_post",
    "Cadera1": "Hip_baseline",
    "Cadera2": "Hip_post",
}
cc_data = cc_data.rename(columns=rename_columns)


# Verified original allocation by row order: 0=Control for rows 31–60; 1=IVR for rows 1–30.
cc_data["Treatment"] = (np.arange(len(cc_data)) < 30).astype(int)
cc_data["BMI_centered"] = cc_data["BMI_baseline"] - cc_data["BMI_baseline"].mean()
cc_data["BMI_centered_sq"] = cc_data["BMI_centered"] ** 2
cc_data["BMI_category_25"] = (cc_data["BMI_baseline"] >= 25).astype(int)


assert len(cc_data) == 60
assert int(cc_data["Treatment"].sum()) == 30
assert int((cc_data["Treatment"] == 0).sum()) == 30
assert int(cc_data.isna().sum().sum()) == 0


bmi_centering_mean = cc_data["BMI_baseline"].mean()
print("DATA PREPARATION")
print(f"N complete-case participants: {len(cc_data)}")
print(f"IVR (Treatment=1): {int(cc_data['Treatment'].sum())}")
print(f"Control (Treatment=0): {int((cc_data['Treatment'] == 0).sum())}")
print(f"Baseline BMI mean used for centering: {bmi_centering_mean:.10f} kg/m²")
print()
```

```
# ==============================================================================
# 3. PRINCIPAL CONTINUOUS TREATMENT-EFFECT MODIFICATION MODELS
# ==============================================================================

linear_formulas = {
    "BMI": "BMI_post ~ Treatment + BMI_centered + Treatment:BMI_centered",
    "Body weight": (
        "Weight_post ~ Weight_baseline + Treatment + BMI_centered + "
        "Treatment:BMI_centered"
    ),
    "Waist circumference": (
        "Waist_post ~ Waist_baseline + Treatment + BMI_centered + "
        "Treatment:BMI_centered"
    ),
    "Hip circumference": (
        "Hip_post ~ Hip_baseline + Treatment + BMI_centered + "
        "Treatment:BMI_centered"
    ),
}

linear_models = {
    name: smf.ols(formula, data=cc_data).fit()
    for name, formula in linear_formulas.items()
}

continuous_results = pd.concat(
    [model_coefficient_table(model, name) for name, model in linear_models.items()],
    ignore_index=True,
```

```
)
print("PRINCIPAL CONTINUOUS MODERATION MODELS")
print(continuous_results.to_string(index=False))
print()


# ==========================================================================
# 4. HOLM ADJUSTMENT FOR SECONDARY CONTINUOUS INTERACTIONS
# ==========================================================================

interaction_term_centered = "Treatment:BMI_centered"
secondary_outcomes = ["Body weight", "Waist circumference", "Hip circumference"]
secondary_raw_p = np.array(
    [linear_models[name].pvalues[interaction_term_centered] for name in secondary_outcomes]
)
_, secondary_holm_p, _, _ = multipletests(secondary_raw_p, method="holm")
holm_results = pd.DataFrame(
    {
        "Outcome": secondary_outcomes,
        "Raw interaction p-value": secondary_raw_p,
        "Holm-adjusted p-value": secondary_holm_p,
    }
)
print("HOLM-ADJUSTED SECONDARY CONTINUOUS INTERACTIONS")
print(holm_results.to_string(index=False))
print()
```

```
# ==========================================================================
# 5. CONDITIONAL IVR–CONTROL DIFFERENCES FOR POST-INTERVENTION BMI
# ==========================================================================

primary_bmi_model = linear_models["BMI"]
primary_parameter_names = list(primary_bmi_model.params.index)
primary_covariance = primary_bmi_model.cov_params().to_numpy()
primary_t_critical = stats.t.ppf(0.975, df=primary_bmi_model.df_resid)

conditional_rows = []
for bmi_value in [22, 27, 32, 37]:
    contrast_vector = np.zeros(len(primary_parameter_names))
    contrast_vector[primary_parameter_names.index("Treatment")] = 1.0
    contrast_vector[primary_parameter_names.index(interaction_term_centered)] = (
        bmi_value - bmi_centering_mean
    )
    estimate = float(contrast_vector @ primary_bmi_model.params.to_numpy())
    standard_error = float(
        np.sqrt(contrast_vector @ primary_covariance @ contrast_vector)
    )
    conditional_rows.append(
        {
            "Baseline BMI": bmi_value,
            "Estimated IVR − Control difference": estimate,
            "SE": standard_error,
            "95% CI lower": estimate - primary_t_critical * standard_error,
            "95% CI upper": estimate + primary_t_critical * standard_error,
        }
```

```
    )
conditional_effects = pd.DataFrame(conditional_rows)
print("CONDITIONAL IVR–CONTROL DIFFERENCES FOR POST-INTERVENTION BMI")
print(conditional_effects.to_string(index=False))
print()


# ============================================================================
# 6. SEX-ADJUSTED SENSITIVITY ANALYSES
# ============================================================================

sex_adjusted_formulas = {
    "BMI": (
        "BMI_post ~ Treatment + BMI_centered + Treatment:BMI_centered + C(Sex)"
    ),
    "Body weight": (
        "Weight_post ~ Weight_baseline + Treatment + BMI_centered + "
        "Treatment:BMI_centered + C(Sex)"
    ),
    "Waist circumference": (
        "Waist_post ~ Waist_baseline + Treatment + BMI_centered + "
        "Treatment:BMI_centered + C(Sex)"
    ),
    "Hip circumference": (
        "Hip_post ~ Hip_baseline + Treatment + BMI_centered + "
        "Treatment:BMI_centered + C(Sex)"
    ),
}
```

```
sex_adjusted_models = {
    name: smf.ols(formula, data=cc_data).fit()
    for name, formula in sex_adjusted_formulas.items()
}
sex_adjusted_rows = []
for name, model in sex_adjusted_models.items():
    row = {"Outcome": name}
    row.update(interaction_result(model, interaction_term_centered))
    sex_adjusted_rows.append(row)
sex_adjusted_results = pd.DataFrame(sex_adjusted_rows)
print("SEX-ADJUSTED SENSITIVITY INTERACTIONS")
print(sex_adjusted_results.to_string(index=False))
print()


# ============================================================================
# 7. MODEL DIAGNOSTICS
# ============================================================================

diagnostic_rows = []
cook_threshold = 4 / len(cc_data)
for name, model in linear_models.items():
    influence = model.get_influence()
    cook_distance = influence.cooks_distance[0]
    leverage = influence.hat_matrix_diag
    studentized_residual = influence.resid_studentized_external
    shapiro_w, shapiro_p = stats.shapiro(model.resid)
    _, breusch_pagan_p, _, _ = het_breuschpagan(model.resid, model.model.exog)
```

```
    exog_without_intercept = pd.DataFrame(
        model.model.exog, columns=model.model.exog_names
    ).drop(columns="Intercept")
    vif_values = [
        variance_inflation_factor(exog_without_intercept.values, index)
        for index in range(exog_without_intercept.shape[1])
    ]
    diagnostic_rows.append(
        {
            "Outcome": name,
            "Shapiro-Wilk p-value": shapiro_p,
            "Breusch-Pagan p-value": breusch_pagan_p,
            "Minimum VIF": min(vif_values),
            "Maximum VIF": max(vif_values),
            "Maximum Cook's distance": np.max(cook_distance),
            "N Cook's D > 4/N": int(np.sum(cook_distance > cook_threshold)),
            "Maximum leverage": np.max(leverage),
            "N |externally studentized residual| > 3": int(
                np.sum(np.abs(studentized_residual) > 3)
            ),
        }
    )
diagnostic_results = pd.DataFrame(diagnostic_rows)
print("MODEL DIAGNOSTICS")
print(diagnostic_results.to_string(index=False))
print()
```

```
# ============================================================================
# 8. HC3 AND COOK'S-DISTANCE EXCLUSION SENSITIVITY ANALYSES
# ============================================================================

sensitivity_rows = []
for name, formula in linear_formulas.items():
    model = linear_models[name]
    confidence_intervals = model.conf_int().loc[interaction_term_centered]
    sensitivity_rows.append(
        {
            "Outcome": name,
            "Analysis": "OLS full sample",
            "N": int(model.nobs),
            "Number excluded": 0,
            **interaction_result(model, interaction_term_centered),
        }
    )

    robust_model = model.get_robustcov_results(cov_type="HC3")
    interaction_index = model.model.exog_names.index(interaction_term_centered)
    robust_confidence_intervals = robust_model.conf_int()[interaction_index]
    sensitivity_rows.append(
        {
            "Outcome": name,
            "Analysis": "HC3 full sample",
            "N": int(model.nobs),
            "Number excluded": 0,
```

```
                "Interaction B": robust_model.params[interaction_index],
                "SE": robust_model.bse[interaction_index],
                "95% CI lower": robust_confidence_intervals[0],
                "95% CI upper": robust_confidence_intervals[1],
                "p-value": robust_model.pvalues[interaction_index],
            }
        )

        outcome_cook_distance = model.get_influence().cooks_distance[0]
        excluded_index = cc_data.index[outcome_cook_distance > cook_threshold]
        cook_exclusion_model = smf.ols(
            formula, data=cc_data.drop(index=excluded_index)
        ).fit()
        sensitivity_rows.append(
            {
                "Outcome": name,
                "Analysis": "OLS excluding Cook's D > 4/N",
                "N": int(cook_exclusion_model.nobs),
                "Number excluded": int(len(excluded_index)),
                **interaction_result(cook_exclusion_model, interaction_term_centered),
            }
        )
    sensitivity_results = pd.DataFrame(sensitivity_rows)
    print("HC3 AND COOK'S-DISTANCE SENSITIVITY ANALYSES")
    print(sensitivity_results.to_string(index=False))
    print()
```

```
# ================================================================================
# 9. EXPLORATORY QUADRATIC NON-LINEARITY ANALYSES
# ================================================================================

quadratic_formulas = {
    "BMI": (
        "BMI_post ~ Treatment + BMI_centered + BMI_centered_sq + "
        "Treatment:BMI_centered + Treatment:BMI_centered_sq"
    ),
    "Body weight": (
        "Weight_post ~ Weight_baseline + Treatment + BMI_centered + BMI_centered_sq + "
        "Treatment:BMI_centered + Treatment:BMI_centered_sq"
    ),
    "Waist circumference": (
        "Waist_post ~ Waist_baseline + Treatment + BMI_centered + BMI_centered_sq + "
        "Treatment:BMI_centered + Treatment:BMI_centered_sq"
    ),
    "Hip circumference": (
        "Hip_post ~ Hip_baseline + Treatment + BMI_centered + BMI_centered_sq + "
        "Treatment:BMI_centered + Treatment:BMI_centered_sq"
    ),
}

quadratic_rows = []
quadratic_models = {}
for name, quadratic_formula in quadratic_formulas.items():
    linear_model = linear_models[name]
    quadratic_model = smf.ols(quadratic_formula, data=cc_data).fit()
```

```
        quadratic_models[name] = quadratic_model
        nested_test = anova_lm(linear_model, quadratic_model)
        quadratic_rows.append(
            {
                "Outcome": name,
                "Linear model AIC": linear_model.aic,
                "Quadratic model AIC": quadratic_model.aic,
                "Linear model adjusted R²": linear_model.rsquared_adj,
                "Quadratic model adjusted R²": quadratic_model.rsquared_adj,
                "Nested F statistic": nested_test.loc[1, "F"],
                "Difference in degrees of freedom": int(nested_test.loc[1, "df_diff"]),
                "Nested F-test p-value": nested_test.loc[1, "Pr(>F)"],
            }
        )
quadratic_results = pd.DataFrame(quadratic_rows)
print("EXPLORATORY QUADRATIC NON-LINEARITY ANALYSES")
print(quadratic_results.to_string(index=False))
print()


# ==========================================================================
# 10. POST HOC EXPLORATORY CATEGORICAL BMI ANALYSES
# ==========================================================================


# The established BMI categorical sensitivity model retains baseline BMI.
# This is necessary to reproduce the verified categorical BMI interaction.
categorical_formulas = {
```

```
    "BMI": "BMI_post ~ BMI_baseline + Treatment + BMI_category_25 + Treatment:BMI_category_25",
    "Body weight": (
        "Weight_post ~ Weight_baseline + Treatment + BMI_category_25 + "
        "Treatment:BMI_category_25"
    ),
    "Waist circumference": (
        "Waist_post ~ Waist_baseline + Treatment + BMI_category_25 + "
        "Treatment:BMI_category_25"
    ),
    "Hip circumference": (
        "Hip_post ~ Hip_baseline + Treatment + BMI_category_25 + "
        "Treatment:BMI_category_25"
    ),
}


categorical_distribution = pd.crosstab(
    cc_data["BMI_category_25"], cc_data["Treatment"]
).rename(columns={0: "Control", 1: "IVR"})
categorical_distribution["Total"] = categorical_distribution.sum(axis=1)
print("POST HOC EXPLORATORY CATEGORICAL ANALYSES: BMI CATEGORY DISTRIBUTION")
print(categorical_distribution.to_string())
print()


categorical_rows = []
for name, formula in categorical_formulas.items():
    categorical_model = smf.ols(formula, data=cc_data).fit()
    categorical_interaction_term = "Treatment:BMI_category_25"
```

```
    row = {"Outcome": name}
    row.update(interaction_result(categorical_model, categorical_interaction_term))
    categorical_rows.append(row)
categorical_results = pd.DataFrame(categorical_rows)
print("POST HOC EXPLORATORY CATEGORICAL BMI INTERACTIONS")
print(categorical_results.to_string(index=False))
print()


# ============================================================================
# 11. MULTIPLE-IMPUTATION SENSITIVITY ANALYSIS: N=64 RANDOMIZED DATASET
# ============================================================================

mi_data = pd.read_excel(RANDOMIZED_FILE, sheet_name=RANDOMIZED_SHEET)
mi_data.columns = [str(column).strip() for column in mi_data.columns]
mi_data["Treatment_num"] = (mi_data["Treatment"] == "IVR").astype(int)
mi_data["Sex_num"] = mi_data["Género"].astype(int)
mi_data["Treatment_x_IMC1"] = mi_data["Treatment_num"] * mi_data["IMC1"]

imputation_predictors = [
    "Treatment_num",
    "Sex_num",
    "IMC1",
    "Peso1",
    "Talla1",
    "Cintura1",
    "Cadera1",
    "Treatment_x_IMC1",
```

```
]
missing_imc2 = mi_data["IMC2"].isna()
observed_imc2 = ~missing_imc2

assert len(mi_data) == 64
assert int((mi_data["Treatment"] == "IVR").sum()) == 32
assert int((mi_data["Treatment"] == "Control").sum()) == 32
assert int(observed_imc2.sum()) == 60
assert int(missing_imc2.sum()) == 4
assert int(((mi_data["Treatment"] == "IVR") & missing_imc2).sum()) == 2
assert int(((mi_data["Treatment"] == "Control") & missing_imc2).sum()) == 2
assert int(mi_data[imputation_predictors].isna().sum().sum()) == 0

# Bayesian linear-regression imputation under MAR.
# Treatment_x_IMC1 is a fixed, fully observed predictor and preserves compatibility
# with the substantive Treatment × IMC1 interaction model.
x_observed = sm.add_constant(
    mi_data.loc[observed_imc2, imputation_predictors], has_constant="add"
).to_numpy(dtype=float)
y_observed = mi_data.loc[observed_imc2, "IMC2"].to_numpy(dtype=float)
x_missing = sm.add_constant(
    mi_data.loc[missing_imc2, imputation_predictors], has_constant="add"
).to_numpy(dtype=float)

xtx_inverse = np.linalg.inv(x_observed.T @ x_observed)
beta_hat = xtx_inverse @ x_observed.T @ y_observed
imputation_residual = y_observed - x_observed @ beta_hat
imputation_residual_df = len(y_observed) - x_observed.shape[1]
```

```
imputation_sse = imputation_residual @ imputation_residual


rng = np.random.default_rng(RANDOM_SEED)
mi_interaction_estimates = []
mi_within_variances = []
observed_imc2_reference = mi_data.loc[observed_imc2, "IMC2"].copy()


for _ in range(N_IMPUTATIONS):
    sigma2_draw = stats.invgamma.rvs(
        a=imputation_residual_df / 2,
        scale=imputation_sse / 2,
        random_state=rng,
    )
    beta_draw = rng.multivariate_normal(
        beta_hat, sigma2_draw * xtx_inverse
    )
    imputed_missing_imc2 = (
        x_missing @ beta_draw
        + rng.normal(0, np.sqrt(sigma2_draw), size=x_missing.shape[0])
    )

    completed_imc2 = mi_data["IMC2"].copy()
    completed_imc2.loc[missing_imc2] = imputed_missing_imc2
    assert completed_imc2.loc[observed_imc2].equals(observed_imc2_reference)

    mi_substantive_design = sm.add_constant(
        pd.DataFrame(
            {
```

```
                "IMC1": mi_data["IMC1"],
                "Treatment": mi_data["Treatment_num"],
                "IMC1_Treatment": mi_data["IMC1"] * mi_data["Treatment_num"],
            }
        ),
        has_constant="add",
    )
    mi_substantive_fit = sm.OLS(
        completed_imc2.to_numpy(dtype=float), mi_substantive_design
    ).fit()
    mi_interaction_estimates.append(mi_substantive_fit.params["IMC1_Treatment"])
    mi_within_variances.append(
        mi_substantive_fit.cov_params().loc["IMC1_Treatment", "IMC1_Treatment"]
    )

# Rubin's rules and Barnard–Rubin small-sample degrees of freedom.
mi_interaction_estimates = np.asarray(mi_interaction_estimates)
mi_within_variances = np.asarray(mi_within_variances)
mi_qbar = float(mi_interaction_estimates.mean())
mi_ubar = float(mi_within_variances.mean())
mi_between_variance = float(mi_interaction_estimates.var(ddof=1))
mi_total_variance = mi_ubar + (1 + 1 / N_IMPUTATIONS) * mi_between_variance
mi_pooled_se = float(np.sqrt(mi_total_variance))
mi_riv = float(
    ((1 + 1 / N_IMPUTATIONS) * mi_between_variance) / mi_ubar
)
mi_old_df = float((N_IMPUTATIONS - 1) * (1 + 1 / mi_riv) ** 2)
mi_complete_data_df = len(mi_data) - mi_substantive_design.shape[1]
```

```
mi_fmi = float((mi_riv + 2 / (mi_old_df + 3)) / (mi_riv + 1))
mi_observed_data_df = float(
    ((mi_complete_data_df + 1) / (mi_complete_data_df + 3))
    * mi_complete_data_df
    * (1 - mi_fmi)
)
mi_pooled_df = float(1 / (1 / mi_old_df + 1 / mi_observed_data_df))
mi_t_statistic = mi_qbar / mi_pooled_se
mi_p_value = float(2 * stats.t.sf(abs(mi_t_statistic), df=mi_pooled_df))
mi_t_critical = float(stats.t.ppf(0.975, df=mi_pooled_df))

mi_results = pd.DataFrame(
    [
        {
            "Pooled interaction B": mi_qbar,
            "Pooled SE": mi_pooled_se,
            "95% CI lower": mi_qbar - mi_t_critical * mi_pooled_se,
            "95% CI upper": mi_qbar + mi_t_critical * mi_pooled_se,
            "p-value": mi_p_value,
            "FMI": mi_fmi,
            "RIV": mi_riv,
            "Pooled df": mi_pooled_df,
            "N": len(mi_data),
            "m": N_IMPUTATIONS,
            "Random seed": RANDOM_SEED,
        }
    ]
)
```

```
print("MULTIPLE-IMPUTATION SENSITIVITY ANALYSIS")
print(mi_results.to_string(index=False))
print()


# ==========================================================================
# 12. FINAL CONTINUOUS MODERATION FIGURES
# ==========================================================================

plt.rcParams.update(
    {
        "font.family": "DejaVu Sans",
        "font.size": 10.5,
        "axes.labelsize": 11,
        "axes.titlesize": 11.5,
        "xtick.labelsize": 10,
        "ytick.labelsize": 10,
    }
)
line_color = "#1F5A8A"
band_color = "#A8C2D6"
reference_color = "#555555"
observed_bmi = cc_data["BMI_baseline"].to_numpy()
bmi_range = np.linspace(observed_bmi.min(), observed_bmi.max(), 400)


def conditional_effect_curve(model, bmi_values):
    """Return IVR−Control effect and conventional 95% CI across baseline BMI."""
```

```
    parameter_names = list(model.params.index)
    covariance = model.cov_params().to_numpy()
    contrast_matrix = np.zeros((len(bmi_values), len(parameter_names)))
    contrast_matrix[:, parameter_names.index("Treatment")] = 1.0
    contrast_matrix[:, parameter_names.index("Treatment:BMI_centered")] = (
        bmi_values - bmi_centering_mean
    )
    effect = contrast_matrix @ model.params.to_numpy()
    variance = np.einsum("ij,jk,ik->i", contrast_matrix, covariance, contrast_matrix)
    standard_error = np.sqrt(variance)
    critical_value = stats.t.ppf(0.975, df=model.df_resid)
    return effect, effect - critical_value * standard_error, effect + critical_value * standard_error


# Main BMI moderation figure.
bmi_effect, bmi_lower, bmi_upper = conditional_effect_curve(
    linear_models["BMI"], bmi_range
)
fig1, ax1 = plt.subplots(figsize=(7.7, 5.35), dpi=600)
ax1.fill_between(bmi_range, bmi_lower, bmi_upper, color=band_color, alpha=0.60, linewidth=0)
ax1.plot(bmi_range, bmi_effect, color=line_color, linewidth=2.2)
ax1.axhline(0, color=reference_color, linestyle=(0, (4, 3)), linewidth=0.9)
axis_ymin, axis_ymax = ax1.get_ylim()
ax1.plot(
    observed_bmi,
    np.full(observed_bmi.shape, axis_ymin + 0.025 * (axis_ymax - axis_ymin)),
    "|",
    color="#5E5E5E",
```

```
        markersize=7,
        markeredgewidth=0.9,
        alpha=0.75,
)
illustrative_bmi = np.array([22, 27, 32, 37], dtype=float)
illustrative_effect = (
        linear_models["BMI"].params["Treatment"]
        + linear_models["BMI"].params["Treatment:BMI_centered"]
        * (illustrative_bmi - bmi_centering_mean)
)
ax1.scatter(
        illustrative_bmi,
        illustrative_effect,
        s=18,
        facecolor="white",
        edgecolor=line_color,
        linewidth=1.15,
        zorder=5,
)
ax1.set_xlim(observed_bmi.min(), observed_bmi.max())
ax1.set_xlabel("Baseline BMI (kg/m²)")
ax1.set_ylabel("Estimated IVR − Control difference in\npost-intervention BMI (kg/m²)")
ax1.grid(axis="y", color="#E5E5E5", linewidth=0.6)
ax1.spines["top"].set_visible(False)
ax1.spines["right"].set_visible(False)
fig1.tight_layout(pad=0.7)
fig1.savefig(OUTPUT_DIR / "Figure1_BMI_moderation.png", dpi=600, bbox_inches="tight")
fig1.savefig(OUTPUT_DIR / "Figure1_BMI_moderation.tiff", dpi=600, bbox_inches="tight")
```

```
plt.close(fig1)


# Four-panel supplementary figure.
supplement_specs = [
    ("BMI", "A. BMI (kg/m²)", "Estimated IVR − Control difference"),
    ("Body weight", "B. Body weight (kg)", "Estimated IVR − Control difference"),
    ("Waist circumference", "C. Waist circumference (cm)", "Estimated IVR − Control difference"),
    ("Hip circumference", "D. Hip circumference (cm)", "Estimated IVR − Control difference"),
]
fig_s1, axes = plt.subplots(2, 2, figsize=(9.2, 7.25), dpi=600, sharex=True)
for axis, (outcome_name, panel_title, y_label) in zip(axes.flat, supplement_specs):
    effect, lower, upper = conditional_effect_curve(linear_models[outcome_name], bmi_range)
    axis.fill_between(bmi_range, lower, upper, color=band_color, alpha=0.60, linewidth=0)
    axis.plot(bmi_range, effect, color=line_color, linewidth=2.0)
    axis.axhline(0, color=reference_color, linestyle=(0, (4, 3)), linewidth=0.85)
    ymin, ymax = axis.get_ylim()
    axis.plot(
        observed_bmi,
        np.full(observed_bmi.shape, ymin + 0.025 * (ymax - ymin)),
        "|",
        color="#5E5E5E",
        markersize=5.1,
        markeredgewidth=0.8,
        alpha=0.7,
    )
    axis.set_title(panel_title, loc="left", fontweight="bold", pad=7)
    axis.set_ylabel(y_label, labelpad=7)
    axis.set_xlim(observed_bmi.min(), observed_bmi.max())
```

```
    axis.grid(axis="y", color="#E5E5E5", linewidth=0.55)

    axis.spines["top"].set_visible(False)

    axis.spines["right"].set_visible(False)

for axis in axes[1, :]:

    axis.set_xlabel("Baseline BMI (kg/m²)")

fig_s1.subplots_adjust(left=0.14, right=0.985, bottom=0.11, top=0.95, wspace=0.36, hspace=0.38)

fig_s1.savefig(OUTPUT_DIR / "Supplementary_Figure_S1_moderation.png", dpi=600, bbox_inches="tight")

fig_s1.savefig(OUTPUT_DIR / "Supplementary_Figure_S1_moderation.tiff", dpi=600, bbox_inches="tight")

plt.close(fig_s1)



# ============================================================================

# 13. FINAL VERIFICATION SUMMARY

# ============================================================================


verification = []


# Data preparation.

check_close("N complete-case", len(cc_data), 60, 0, verification)

check_close("IVR allocation", int(cc_data["Treatment"].sum()), 30, 0, verification)

check_close("Control allocation", int((cc_data["Treatment"] == 0).sum()), 30, 0, verification)

check_close("Mean baseline BMI", bmi_centering_mean, 26.2971581148, 1e-8, verification)


# Continuous interaction model checks.

continuous_expected = {

    "BMI": (-0.03894663, 0.02928663, 0.18896130, 0.98643320, 56),

    "Body weight": (-0.08844037, 0.08253983, 0.28862978, 0.99313655, 55),
```

```
    "Waist circumference": (-0.02856380, 0.07381565, 0.70027810, 0.98910187, 55),
    "Hip circumference": (-0.12949290, 0.13079622, 0.32649247, 0.88922300, 55),
}
for outcome_name, (expected_b, expected_se, expected_p, expected_adj_r2, expected_df) in continuous_expected.items():
    fitted_model = linear_models[outcome_name]
    check_close(f"{outcome_name}: interaction B", fitted_model.params[interaction_term_centered], expected_b, 1e-6, verification)
    check_close(f"{outcome_name}: interaction SE", fitted_model.bse[interaction_term_centered], expected_se, 1e-6, verification)
    check_close(f"{outcome_name}: interaction p", fitted_model.pvalues[interaction_term_centered], expected_p, 1e-6, verification)
    check_close(f"{outcome_name}: adjusted R²", fitted_model.rsquared_adj, expected_adj_r2, 1e-6, verification)
    check_close(f"{outcome_name}: residual df", fitted_model.df_resid, expected_df, 0, verification)


# Holm results.
for outcome_name, actual_value in zip(secondary_outcomes, secondary_holm_p):
    check_close(f"{outcome_name}: Holm-adjusted p", actual_value, 0.86588935, 1e-6, verification)


# Conditional effects.
conditional_expected = {
    22: (-0.09169258, 0.18044839),
    27: (-0.28642575, 0.13300272),
    32: (-0.48115892, 0.21378683),
    37: (-0.67589209, 0.34146614),
}
for _, row in conditional_effects.iterrows():
    expected_effect, expected_se = conditional_expected[int(row["Baseline BMI"])]
    check_close(f"BMI {int(row['Baseline BMI'])}: conditional effect", row["Estimated IVR − Control difference"], expected_effect, 1e-5, verification)
```

```
    check_close(f"BMI {int(row['Baseline BMI'])}: conditional SE", row["SE"], expected_se, 1e-5, verification)


# Sex-adjusted sensitivity checks.
sex_expected = {
    "BMI": (-0.03864772, 0.03018599, 0.205875),
    "Body weight": (-0.08198126, 0.08504215, 0.339334),
    "Waist circumference": (-0.02205410, 0.07652209, 0.774295),
    "Hip circumference": (-0.05285892, 0.12636742, 0.677406),
}
for _, row in sex_adjusted_results.iterrows():
    expected_b, expected_se, expected_p = sex_expected[row["Outcome"]]
    check_close(f"{row['Outcome']}: sex-adjusted B", row["Interaction B"], expected_b, 1e-5, verification)
    check_close(f"{row['Outcome']}: sex-adjusted SE", row["SE"], expected_se, 1e-5, verification)
    check_close(f"{row['Outcome']}: sex-adjusted p", row["p-value"], expected_p, 1e-5, verification)


# Diagnostics.
diagnostic_expected = {
    "BMI": (0.00178183, 0.15548727, 0.10112659, 6, 0.37524122, 2),
    "Body weight": (0.00011936, 0.32595866, 0.09193285, 4, 0.38104040, 2),
    "Waist circumference": (0.00000177, 0.08482447, 0.09817355, 2, 0.37629914, 2),
    "Hip circumference": (0.00000000908, 0.33590882, 0.20335288, 4, 0.37709814, 2),
}
for _, row in diagnostic_results.iterrows():
    expected_shapiro, expected_bp, expected_cook, expected_n_cook, expected_lev, expected_n_stud = diagnostic_expected[row["Outcome"]]
    check_close(f"{row['Outcome']}: Shapiro p", row["Shapiro-Wilk p-value"], expected_shapiro, 1e-6, verification)
```

```
check_close(f"{row['Outcome']}: BP p", row["Breusch-Pagan p-value"], expected_bp, 1e-6, verification)

check_close(f"{row['Outcome']}: max Cook", row["Maximum Cook's distance"], expected_cook, 1e-6, verification)

check_close(f"{row['Outcome']}: N Cook", row["N Cook's D > 4/N"], expected_n_cook, 0, verification)

check_close(f"{row['Outcome']}: max leverage", row["Maximum leverage"], expected_lev, 1e-6, verification)

check_close(f"{row['Outcome']}: N studentized", row["N |externally studentized residual| > 3"], expected_n_stud, 0, verification)


# HC3 and Cook-exclusion interaction p-values.

sensitivity_expected_p = {

    ("BMI", "HC3 full sample"): 0.13210065,

    ("BMI", "OLS excluding Cook's D > 4/N"): 0.07086948,

    ("Body weight", "HC3 full sample"): 0.17233542,

    ("Body weight", "OLS excluding Cook's D > 4/N"): 0.03073037,

    ("Waist circumference", "HC3 full sample"): 0.47752988,

    ("Waist circumference", "OLS excluding Cook's D > 4/N"): 0.82770307,

    ("Hip circumference", "HC3 full sample"): 0.39369965,

    # The manuscript-facing value is approximately 0.016; the unrounded OLS result is used for verification.

    ("Hip circumference", "OLS excluding Cook's D > 4/N"): 0.01585647,

}

for (outcome_name, analysis_name), expected_p in sensitivity_expected_p.items():

    selected_row = sensitivity_results.loc[

        (sensitivity_results["Outcome"] == outcome_name)

        & (sensitivity_results["Analysis"] == analysis_name)

    ].iloc[0]

    check_close(f"{outcome_name}: {analysis_name} p", selected_row["p-value"], expected_p, 1e-5, verification)
```

```
# Quadratic comparisons.
quadratic_expected_p = {
    "BMI": 0.17452066,
    "Body weight": 0.38466868,
    "Waist circumference": 0.97806479,
    "Hip circumference": 0.03012102,
}
for _, row in quadratic_results.iterrows():
    check_close(f"{row['Outcome']}: quadratic F-test p", row["Nested F-test p-value"], quadratic_expected_p[row["Outcome"]], 1e-6, verification)


# Categorical interactions.
categorical_expected = {
    "BMI": (-0.67757099, 0.01055789),
    "Body weight": (-1.71085519, 0.01990778),
    "Waist circumference": (-0.72923533, 0.26508042),
    "Hip circumference": (-2.16088932, 0.04951289),
}
for _, row in categorical_results.iterrows():
    expected_b, expected_p = categorical_expected[row["Outcome"]]
    check_close(f"{row['Outcome']}: categorical B", row["Interaction B"], expected_b, 1e-5, verification)
    check_close(f"{row['Outcome']}: categorical p", row["p-value"], expected_p, 1e-5, verification)


# Multiple imputation.
check_close("MI pooled interaction B", mi_qbar, -0.03857, 1e-5, verification)
check_close("MI pooled SE", mi_pooled_se, 0.02924, 1e-5, verification)
check_close("MI pooled p", mi_p_value, 0.192389, 1e-6, verification)
```

```
check_close("MI FMI", mi_fmi, 0.00707, 1e-5, verification)
check_close("MI RIV", mi_riv, 0.00712, 1e-5, verification)
check_close("MI pooled df", mi_pooled_df, 57.68091, 1e-4, verification)


verification_df = pd.DataFrame(verification)
verification_df.to_csv(OUTPUT_DIR / "verification_summary.csv", index=False)


print("FINAL VERIFICATION SUMMARY")
print(verification_df.to_string(index=False))
if (verification_df["Status"] == "VERIFICATION FAILED").any():
    print("\nVERIFICATION FAILED")
    print(
        verification_df.loc[
            verification_df["Status"] == "VERIFICATION FAILED"
        ].to_string(index=False)
    )
else:
    print("\nAll requested analytical results reproduced within the prespecified numerical tolerances.")


# Save compact tables produced by the script.
continuous_results.to_csv(OUTPUT_DIR / "continuous_models.csv", index=False)
holm_results.to_csv(OUTPUT_DIR / "holm_interactions.csv", index=False)
conditional_effects.to_csv(OUTPUT_DIR / "conditional_bmi_effects.csv", index=False)
sex_adjusted_results.to_csv(OUTPUT_DIR / "sex_adjusted_interactions.csv", index=False)
diagnostic_results.to_csv(OUTPUT_DIR / "model_diagnostics.csv", index=False)
sensitivity_results.to_csv(OUTPUT_DIR / "hc3_cook_sensitivity.csv", index=False)
quadratic_results.to_csv(OUTPUT_DIR / "quadratic_model_comparisons.csv", index=False)
```

```
categorical_results.to_csv(OUTPUT_DIR / "categorical_bmi_interactions.csv", index=False)

mi_results.to_csv(OUTPUT_DIR / "multiple_imputation_results.csv", index=False)
```

CONSORT CHECK LIST

	Item Description	Location (or reason for not reporting)
**Title and Abstract**		
1a. Title	Identification as a randomised trial.	Page 1
1b. Structured Abstract	Structured summary of the trial design, methods, results, and conclusions.	Page 2
**Open Science**		
2. Trial Registration	Name of trial registry, identifying number (with URL) and date of registration.	Page 5
Protocol and statistical analysis plan	Where the trial protocol and statistical analysis plan can be accessed.	Page 5
4. Data sharing	Where and how the individual de-identified participant data (including data dictionary), statistical code and any other materials can be accessed.	Page 18
5. Funding and Conflicts of Interest		
5a. Funding	Sources of funding and other support (eg, supply of drugs), and role of funders in the design, conduct, analysis, and reporting of the trial.	Page 18
5b. Conflicts of interest	Financial and other conflicts of interest of the manuscript authors.	Page 18
**Introduction**		
6. Background and rationale	Scientific background and rationale.	Pages 2 and 3
7. Objectives	Specific objectives related to benefits and harms.	Page 3
**Methods**		
8. Patient and public involvement	Details of patient or public involvement in the design, conduct and reporting of the trial.	Page 5
9. Trial Design	Description of trial design including type of trial (eg, parallel group, crossover), allocation ratio, and framework (eg, superiority, equivalence, non-inferiority, exploratory).	Page 4
10. Changes to trial protocol	Important changes to the trial after it commenced including any outcomes or analyses that were not pre-specified, with reason.	Page 4

11. Trial Setting	Settings (eg, community, hospital) and locations (eg, countries, sites) where the trial was conducted.	Page 4
12. Eligibility Criteria		
12a. Participants	Eligibility criteria for participants.	Pages 5 and 6
12b. Other	If applicable, eligibility criteria for sites and for individuals delivering the interventions (eg, surgeons, physiotherapists).	N/A
13. Intervention and comparator	Intervention and comparator with sufficient details to allow replication. If relevant, where additional materials describing the intervention and comparator (eg, intervention manual) can be accessed.	Pages 6 and 7
14. Outcomes	Prespecified primary and secondary outcomes, including the specific measurement variable (eg, systolic blood pressure), analysis metric (eg, change from baseline, final value, time to event), method of aggregation (eg, median, proportion), and time point for each outcome.	Page 8
15. Harms	How harms were defined and assessed (eg, systematically, non-systematically).	Page 7
16. Sample Size		
16a. How sample size was determined	How sample size was determined, including all assumptions supporting the sample size calculation.	Page 5
16b. Interim analyses and stopping criteria	Explanation of any interim analyses and stopping guidelines.	Page 7
17. Randomisation		
17a. Sequence Generation	Who generated the random allocation sequence and the method used.	Page 7
17b. Type of Randomisation	Type of randomisation and details of any restriction (eg, stratification, blocking, and block size).	Page 7
18. Allocation concealment mechanism	Mechanism used to implement the random allocation sequence (eg, central computer/telephone; sequentially numbered, opaque, sealed containers), describing any steps to conceal the sequence until interventions were assigned.	Page 7

19. Implementation	Whether the personnel who enrolled and those who assigned participants to the interventions had access to the random allocation sequence.	Page 7
20. Blinding		
20a. Who was blinded	Who was blinded after assignment to interventions (eg, participants, care providers, outcome assessors, data analysts).	Page 7
20b. How blinding was achieved	If blinded, how blinding was achieved and description of the similarity of interventions.	Page 7
21. Statistical methods		
21a. Comparing groups	Statistical methods used to compare groups for primary and secondary outcomes, including harms.	Pages 9 and 10
21b. Definition of who is included in each analysis	Definition of who is included in each analysis (e.g., all randomised participants), and in which group.	Pages 9 and 10
21c. Missing Data	How missing data were handled in the analysis.	Pages 9 and 10
21d. Additional Analyses	Methods for any additional analyses (eg, subgroup and sensitivity analyses), distinguishing pre-specified from post hoc.	Pages 9 and 10
22. Participant flow, including flow diagram		
22a. Participant Numbers	For each group, the numbers of participants who were randomly assigned, received intended intervention, and were analysed for the primary outcome.	Page 5
22b. Losses and exclusions	For each group, losses and exclusions after randomisation, together with reasons.	Page 5
23. Recruitment		
23a. Dates	Dates defining the periods of recruitment and follow-up for outcomes of benefits and harms.	Page 5
23b. Reasons for stopping recruitment	If relevant, why the trial ended or was stopped.	Page 5
24. Intervention and comparator delivery		
24a. As Administered	Intervention and comparator as they were actually administered (eg, where appropriate, who	Page 10

	delivered the intervention/comparator, whether participants adhered, whether they were delivered as intended (fidelity)).	
24b. Concomitant Care	Concomitant care received during the trial for each group.	Page 10
25. Baseline Data	A table showing baseline demographic and clinical characteristics for each group.	Page 11
26. Numbers analysed, outcomes, and estimation	For each primary and secondary outcome, by group:<br>• the number of participants included in the analysis.<br>• the number of participants with available data at the outcome time point.<br>• result for each group, and the estimated effect size and its precision (such as 95% confidence interval).<br>• for binary outcomes, presentation of both absolute and relative effect size.	Page 11
27. Harms	All harms or unintended events in each group.	Page 12
28. Ancillary Analyses	Any other analyses performed, including subgroup and sensitivity analyses, distinguishing pre-specified from post hoc.	Page 12
**Discussion**		
29. Interpretation	Interpretation consistent with results, balancing benefits and harms, and considering other relevant evidence.	Pages 14 and 15
30. Limitations	Trial limitations, addressing sources of potential bias, imprecision, generalisability, and, if relevant, multiplicity of analyses.	Page 16
